# Early stages of speciation with gene flow in the Amazilia Hummingbird (*Amazilis amazilia*) subspecies complex of Western South America

**DOI:** 10.1002/ece3.8895

**Published:** 2022-05-13

**Authors:** Sarah A. Cowles, Christopher C. Witt, Elisa Bonaccorso, Felix Grewe, J. Albert C. Uy

**Affiliations:** ^1^ 5452 Department of Biology University of Miami Coral Gables Florida USA; ^2^ Department of Biology and Museum of Southwestern Biology University of New Mexico Albuquerque New Mexico USA; ^3^ Laboratorio de Biología Evolutiva, Colegio de Ciencias Biológicas y Ambientales Universidad San Francisco de Quito Quito Ecuador; ^4^ Centro de Investigación de la Biodiversidad y Cambio Climático Universidad Tecnológica Indoamérica Quito Ecuador; ^5^ Grainger Bioinformatics Center Field Museum Chicago Illinois USA; ^6^ 6927 Department of Biology University of Rochester Rochester New York USA

**Keywords:** *Amazilia*, *Amazilis*, hummingbird, hybridization, phylogeography, speciation, subspecies

## Abstract

Disentangling the factors underlying the diversification of geographically variable species with a wide geographical range is essential to understanding the initial stages and drivers of the speciation process. The Amazilia Hummingbird, *Amazilis amazilia*, is found along the Pacific coast from northern Ecuador down to the Nazca Valley of Peru, and is currently classified as six phenotypically differentiated subspecies. We aimed to resolve the evolutionary relationships of the six subspecies, to assess the geographical pattern and extent of evolutionary divergence, and to test for introgression using both a mtDNA marker and a genome‐by‐sequencing dataset from 86 individuals from across the species range. The consensus phylogenetic tree separated the six subspecies into three distinct clades, corresponding with the Ecuador lowlands (*A*. *amazilia dumerilii*), the Ecuador highlands (*A*. *amazilia alticola* and *A*.* amazilia azuay*), and the Peruvian coast (*A*. *amazilia leucophoea*, *A*. *amazilia amazilia*, and *A*.* amazilia caeruleigularis*). However, an unresolved mtDNA network suggests that the diversification of the subspecies was recent and rapid. We found evidence of gene flow among the subspecies *A*. *amazilia dumerilii*, *A*. *amazilia alticola*, and *A*. *amazilia leucophoea*, with strong genetic isolation of the subspecies *A*. *amazilia azuay* in the isolated Yunguilla Valley of Ecuador. Finally, environmental data from each subspecies’ capture locations were concordant with the three distinct clades. Overall, our results suggest that both expansions into new habitats and geographic isolation shaped the present‐day phylogeny and range of the *A*. *amazilia* subspecies, and that *A*. *amazilia azuay* may be genetically divergent enough to be considered a separate species.

## Graphical abstract

We conducted phylogenomic analyses of 86 Amazilia Hummingbird genetic samples using GBS and mtDNA. We found that the six subspecies are split into three distinct clades (Ecuador highlands, lowlands, and Peru), with evidence of gene flow across subspecies, but strong genetic isolation of the subspecies *azuay* in Ecuador. Climate data from subspecies locations is concordant with the three clades, suggesting both range expansion and geographic isolation likely drove subspecies diversification.

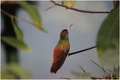

## 1 INTRODUCTION

Many factors can shape the evolutionary trajectory of a species. Environmental features are particularly important in divergence, as they can inhibit or facilitate gene flow, lead to ecological specialization, and affect community structure (e.g., Coyne & Orr, 2004; Dobzhansky, 1937; Graham et al., 2009; McNew et al., 2021; Nosil, 2012; Price, 2008; Rundle & Nosil, 2005). The Andes are the longest above‐sea mountain chain in the world, extending along the entire western margin of South America. This massive ridge is thought to be an engine for biotic diversification because it creates heterogeneity and isolation among habitats and climate regimes (e.g., Benham & Witt, 2016; Fjeldså et al., 2012; Luebert & Weigend, 2014). Pleistocene glacial cycles are thought to have further accelerated diversification in the Andes, as the contraction of montane habitats along elevational gradients during warming periods promoted isolation, with subsequent expansion of montane habitats during cooling allowing dispersal into new areas (Hooghiemstra et al., 2000). As a result, many rapid, recent radiations are associated with the topography of the Andes, in both plants (e.g., Hughes & Eastwood, 2006; Pérez‐Escobar et al., 2017) and animals (e.g., Beckman & Witt, 2015; Elias et al., 2009; Hutter et al., 2013; Weir & Price, 2011).

Within the Americas, the hummingbird family (Trochilidae) has undergone a massive diversification, radiating into 351 species of hummingbirds that are classified into nine major clades (Bleiweiss et al., 1997; Clements et al., 2021; McGuire et al., 2014). Much of this family's diversity has been shaped by landscape features such as the Andean uplift or the formation of the Panamanian land bridge. These geographical features allowed for isolation and provided a variety of niche habitats across elevational gradients, which aided in the diversification of this lineage (Graham et al., 2009; McGuire et al., 2014). Hummingbirds invaded the lowlands of South America approximately 22 million years ago, and certain clades subsequently diversified in the Andes (McGuire et al., 2014). Approximately 40% of all hummingbird species are now found within the Andes (McGuire et al., 2014).

Net diversification rates vary among clades in hummingbirds, making them an ideal system to study the processes that drive speciation. Several clades, including bee hummingbirds (Mellisugini), mountain gems (Lampornithini), and emeralds (Trochilini), show high net diversification rates, which are likely tied to expansion into new South American ranges (McGuire et al., 2014). The clade of emerald hummingbirds (Trochilini) contains 108 species and has evolved within the last 10–15 million years (McGuire et al., 2014). Within the emerald clade is the *Amazilia* group and closely related species (previously known as the *Amazilia* genus, now reclassified into 10 different genera as of Clements et al., 2021), which contains 31 species of medium‐sized hummingbirds that are distributed from the Southern USA to Southern Argentina. This group is thought to have originated west of the Isthmus of Tehuantepec and diversified into two major clades, with one clade spreading east of the isthmus and another clade spreading into South America, with most diversification occurring between the late Miocene and Pliocene (11.6–2.6 million years ago; McGuire et al., 2014; Ornelas et al., 2014). Previous studies have evaluated phylogeography, hybridization, and drivers of diversification in this group across Central and South America using mtDNA and microsatellite markers (e.g., Jiménez & Ornelas, 2016; Miller et al., 2011; Ornelas et al., 2014; Rodríguez‐Gómez & Ornelas, 2013, 2014, 2015, 2018; Rodríguez‐Gómez et al., 2021). These studies have generally found that this group consists of young, rapidly diversifying lineages with hybridization and introgression in zones of secondary contact after initial divergence due to isolation. However, no studies of this group have yet used genome‐scale data to examine the diversification of a variable subspecies complex.

**FIGURE 1:**
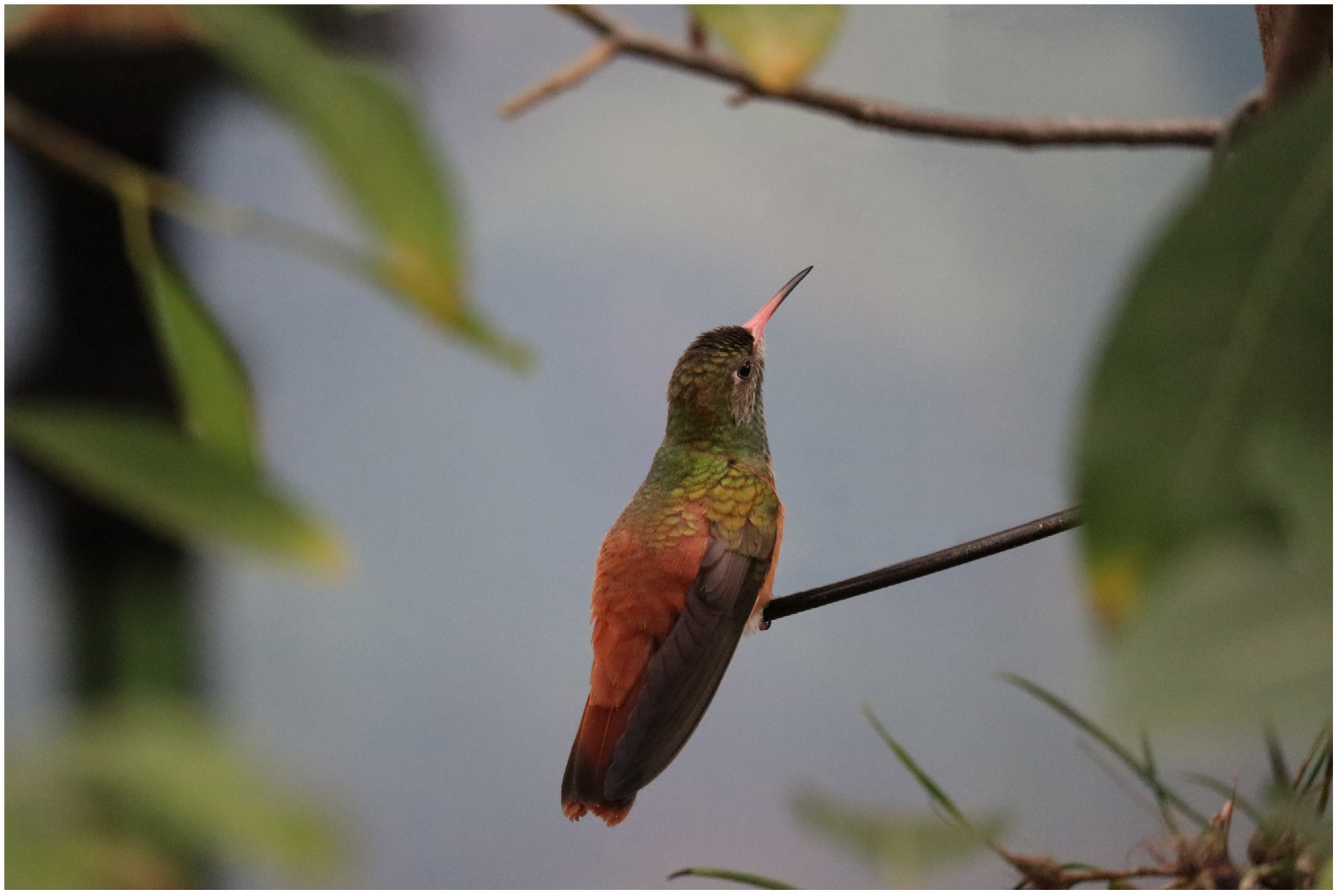
*Amazilis amazilia*. Photo by Felix Grewe

**FIGURE 2:**
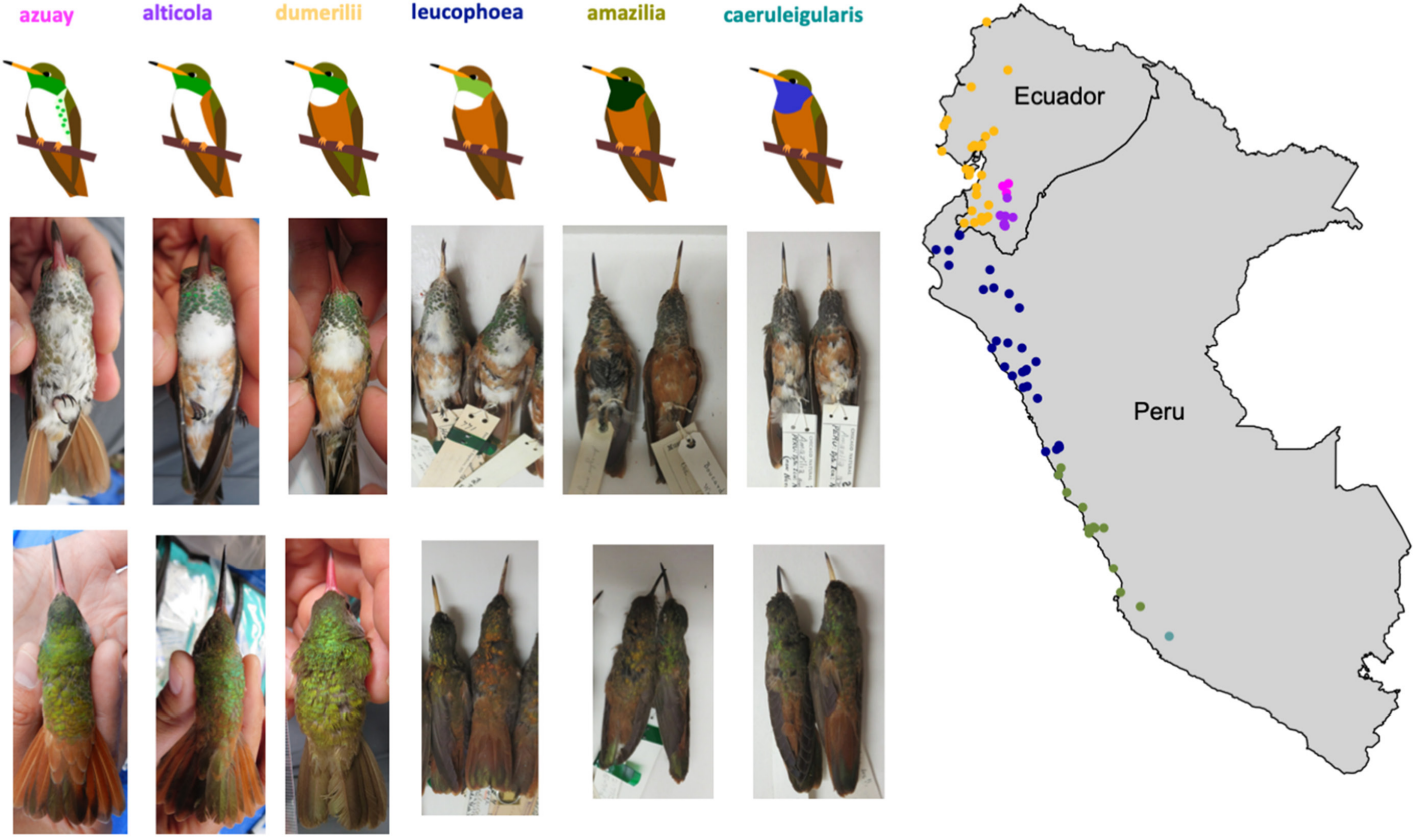
Plumage patterns and range of the six subspecies of *Amazilis amazilia* across Ecuador and Peru. Points on the map indicate historical collection records (from Weller, 2000) as well as our field collection sites (see Table S1)

The Amazilia Hummingbird (*Amazilis amazilia*; previously *Amazilia amazilia* prior to Clements et al., 2021; Figure 1) is a species of medium‐sized hummingbird (9–10 cm, 4–7 g) found along the western coast of Ecuador from close to the Ecuadorian‐Colombian border south to the Nazca Valley in Peru. These hummingbirds inhabit arid and semi‐arid lowland scrub/dry forest environments along the Pacific coast and can also range up into the subtropical forest on the Andean slopes to elevations of up to 2800 m. In addition, they can be found within the gardens of towns and cities, such as Lima and Guayaquil (Calviño‐Cancela, 2006; Weller et al., 2019). *Amazilis amazilia* feeds on nectar from flowers of medium corolla length such as *Salvia splendens*, *Justicia brandegeana*, *Erythrina*, *Psittacanthus*, and *Leonotis nepetifolia*, as well as on small insects that are caught aerially (Calviño‐Cancela, 2006; Weller et al., 2019, S. Cowles pers. obs.). This species is also territorial against conspecifics, other species of hummingbirds, and other nectar feeders such as bananaquits (*Coereba flaveola*) (Calviño‐Cancela, 2006, S. Cowles pers. obs.), and may show small‐range altitudinal migrations following food sources across seasons (Weller et al., 2019, S. Cowles pers. obs.). The current classification recognizes six distinct subspecies (Krabbe & Ridgely, 2010)—three in Ecuador: *A*. *amazilia alticola*, *A*. *amazilia azuay*, and *A*. *amazilia dumerilli*, and three in Peru: *A*. *amazilia leucophoea*, *A*. *amazilia amazilia*, and *A*. *amazilia caeruleigularis* (Figure 2; from here onward subspecies will be written only using the subspecies designation, i.e., *alticola*, *azuay*, *dumerilii*, *leucophoea*, *amazilia*, and *caeruleigularis*). These subspecies differ remarkably in several phenotypic characteristics, such as the presence or absence of white throat patches, rufous belly coloration, tail coloration, and gorget coloration (Figure 2; Krabbe & Ridgely, 2010; Weller, 2000), making them ideal to study the potential factors that underlie divergence in the early stages of the speciation process.

Previous research based on phenotypic similarity (morphology and plumage) has suggested that the northern (*dumerilii* and *leucophoea*) and southern (*amazilia* and *caeruleigularis*) subspecies of *A*. *amazilia* form separate clades (Weller, 2000). However, the evolutionary relationships and history of the higher altitude subspecies in Ecuador (*alticola* and *azuay*) in relation to these possible northern and southern clades remain unknown, and there is debate on whether the subspecies *alticola*, known as the Loja Hummingbird, should be elevated to species status (Krabbe & Ridgely, 2010; Weller, 2000). In addition, little is known about the level of hybridization or intergradation between the six subspecies of *A*. *amazilia* across its range.

This study aimed to first examine the phylogenetic relationships of all six subspecies of *A*. *amazilia*, and then to assess the level of gene flow and population structuring across the six subspecies throughout their ranges using a variety of analytical techniques. In our analyses, we used genomic DNA isolated from field‐collected blood samples and museum tissue specimens to create a genome‐wide dataset of single nucleotide polymorphisms (SNPs) and one mitochondrial gene alignment. In addition, using climate data from each subspecies’ range, we explore geographical boundaries and ecological barriers that potentially led to the subspecies diversification within the *A*. *amazilia* group. We hypothesized that the subspecies *alticola*, *dumerilii*, and *leucophoea* are more genetically similar to one another due to gene flow, given their phenotypic similarity and intergradation, along with larger ranges and the possibility for range overlap in Southern Ecuador and Northern Peru. In contrast, we hypothesized that the subspecies *azuay* in the Yunguilla Valley of Ecuador and *amazilia* and *careuleigularis* in southern coastal Peru are more genetically isolated due to their restricted ranges.

## 2 MATERIAL AND METHODS

### 2.1 Field and DNA extraction methods

We conducted fieldwork from May through July of 2016 to capture *A*. *amazilia* in Ecuador. We captured birds from six different field sites around the range of the three Ecuadorian subspecies (*alticola*, *azuay*, and *dumerilii*; Table S1). To catch birds, we used both 6‐ and 12‐m mist nets in flyways and close to nectar sources, and occasionally used red nectar feeders filled with sugar water as bait. Once captured, we took blood samples from the medial metatarsal vein of each hummingbird. We also clipped an outer right tail retrix to identify the individual in case of recapture. Each bird was given sugar‐water *ad libitum* and subsequently released. Over our field season, we captured a total of 55 *A*. *amazilia* across our six locations in Ecuador (Table S1; *alticola*: 19, *azuay*: 13, *dumerilli*: 23). All field methods were approved beforehand by the University of Miami's Institutional Animal Care and Use Committee (for research permits see Acknowledgments).

In addition to capturing *A*. *amazilia*, we also captured individuals of two closely related species at two field sites: the Rufous‐tailed Hummingbird (*Amazilia tzacatl*, 9 samples) and the Andean Emerald (*Uranomitra franciae*, 2 samples) (Table S1). We used samples from these two species as outgroups in our subsequent phylogenetic analyses. We extracted DNA from all *A*. *amazilia* and closely related species samples using Qiagen's DNeasy Blood and Tissue Extraction Kits (Qiagen, Hilden, Germany) using the manufacturer's protocol for nucleated blood.

We obtained 34 frozen tissue samples of *A*. *amazilia* from the Museum of Southwestern Biology at the University of New Mexico (Table S1; *amazilia*: *9*, *leucophoea*: 21, *caeruleigularis*: 4) that spanned the species range in Peru (West of the Andes from Tumbes/Piura down to the Nazca Valley). We extracted DNA using Qiagen's DNeasy Blood and Tissue Extraction Kits (Qiagen, Hilden, Germany) using the manufacturer's protocol for tissue samples, with the suggested addition of 4 μl of RNase A per sample after tissue digestion to reduce the possibility of any RNA contamination.

### 2.2 Mitochondrial sequencing and analysis

Our first aim was to sequence the mitochondrial gene NADH dehydrogenase subunit 2 (*ND2*). We amplified a fragment of the gene with the L5215 and H1064 primers (Sorenson et al., 1999) using Sorenson et al. (1999)’s standard PCR protocol with an annealing temperature of 54°C. We cleaned the PCR‐amplified DNA using ExoSAP‐IT according to the manufacturer (USB Corporation), and prepared the DNA for sequencing using the BigDyeTerminator Version 3.1 Cycle Sequencing Kit (Applied Biosystems). DNA was purified before sequencing using Sephadex columns (Sigma–Aldrich) and was loaded onto 96‐well plates. Plates were sequenced using Sanger Sequencing in the Institute of Biotechnology at Cornell University (Ithaca, NY, USA). Mitochondrial sequences were then trimmed, inspected, and aligned using Sequencher version 5.4.6 (Gene Codes). Assignment of codon positions and translation into amino acids was conducted in Mesquite 3.2 (Maddison & Maddison, 2017) after aligning novel sequences to sequences of *Amazilia* and related hummingbirds available on GenBank (see Appendix Table 2 in S2). Outgroup sequences were designated based on previous phylogenetic analyses (McGuire et al., 2014).

We used the software program PopART version 1.7 (Leigh & Bryant, 2015) to create a median‐joining haplotype network for all samples of *A*. *amazilia*. Before further phylogenetic analyses, we estimated the best‐fit model of nucleotide substitution for the whole gene in PartitionFinder 2 (Lanfear et al., 2012), using branch lengths linked, greedy algorithm, and Akaike Information Criterion for model selection. To estimate the time frame for the diversification of *ND2* lineages of *A*. *amazilia and* closely related species, we obtained a series of maximum clade credibility trees in BEAST 2.6.6 (Bouckaert et al., 2014). For these analyses, we used the best‐fit model of nucleotide substitution obtained by Partition Finder 2 (i.e., GTR + G). To find the appropriate molecular clock model for the analysis, we applied a Bayes Factor (BF) analysis to compare the marginal likelihoods of trees obtained with a strict clock model and a relaxed log‐normal model. The Log‐marginal likelihoods (Log‐MLs) were estimated with Nested Sampling (Skilling, 2006), an efficient computation algorithm implemented in BEAST 2 (Russel et al., 2019), using 10 particle counts and 10,000 sub chain length. We also compared analyses using different Yule and birth‐death model priors, as well as site models (GTR + G vs. HKY + G), by evaluating the Markov chain Monte Carlo (MCMC) chain convergence and Effective Sample Sizes (ESSs) of model statistics in Tracer 1.7.2 (Rambaut et al., 2018). Also, based on our RaxML likelihood analysis (see Results), we used a monophyly prior for all samples of *A*. *amazilia*, in all analyses. Once the model was optimized, we applied three different clock rates: 0.029 substitutions/site/million year (s/s/million years) (Lerner et al., 2011), 0.0125 s/s/million years (Smith & Klicka, 2010), and the unconventional (slow) clock rate of 0.0068 s/s/million years of Ornelas et al. (2014). These three separate analyses were run for 30 million generations, sampling every 100 generations. Using TreeAnnotator 2.6.6 (Rambaut & Drummond, 2019), we discarded the first 20% of trees as burn‐in and obtained a maximum clade credibility tree with the remaining trees, which was then visualized in FigTree 1.4.3 (Rambaut, 2017).

### 2.3 Genomic sequencing

Our second aim was to obtain thousands of SNPs across the genome using the reduced representation sequencing method of genotyping‐by‐sequencing (GBS) (Elshire et al., 2011). We chose 86 of our 89 *A*. *amazilia* samples based on extracted DNA concentrations (above 5.65 ng/ul; all but MSB:Bird:34693, MSB:Bird:32901, and MSB:Bird:43305), 7 *A*. *tzacatl* (3 from Mindo and 4 from Ayampe), and the 2 *U*. *franciae* samples to fill a 96‐well plate (with one blank well as a control). We first checked extracted genomic DNA quality by running 100 ng of each sample on a 1% agarose gel to confirm the presence of intact, non‐fragmented genomic DNA. We then digested 10% of the samples using the manufacturer's EcoRI digestion protocol (Promega) to confirm the presence of enzymatic activity. We loaded samples onto a 96‐well plate and sent the plate to the University of Wisconsin–Madison's Bioinformatics Resource Center for GBS using the restriction enzyme ApeKI. SNPs were called by the University of Wisconsin–Madison's Bioinformatics Resource Center using the established UNEAK‐TASSEL pipeline and parameters (Glaubitz et al., 2014). This UNEAK‐TASSEL pipeline calls SNPs with a minimum minor allele frequency of 0.05, a minimum count of 5, a mismatch rate of below 0.03, and a minimum call rate of 0.1. This preliminary filtering resulted in a dataset of 1,032,375 SNPs. We additionally filtered the dataset using the filtering function in TASSEL version 1.5 (Bradbury et al., 2007) by choosing only SNPs that had a minor allele frequency of at least 0.05 and that were found in at least 60 of the 95 sequenced individuals. This reduced dataset ultimately included 34,896 SNPs, and we created a vcf file of these SNPs in TASSEL version 1.5 (Bradbury et al., 2007) to use in our downstream analyses.

### 2.4 Genomic analyses

To obtain estimates of population structuring, genetic distance, and gene flow across the six subspecies of *A*. *amazilia*, we used several genomics programs and methods designed to work with genomic SNP data. To reconstruct a phylogeny, we first converted our vcf file of 34,896 SNPs into an interleaved phylip file in TASSEL version 1.5 (Bradbury et al., 2007). We then used the program RAxML version 8.2.8 (Stamatakis, 2014) to build a maximum likelihood tree of the 86 *A*. *amazilia* samples and the two closely related species (7 *A*. *tzacatl* and 2 *U*. *franciae* samples). We used GTRGAMMA (the general time‐reversal model with gamma correction) and the rapid bootstrap option (Stamatakis et al., 2008) to replicate 100 trees. The resulting phylogenetic tree was then drawn to scale in MEGA version 7.0 (Kumar et al., 2016) with the *U*. *franciae* and *A*. *tzacatl* samples specified as outgroups. For a coalescent‐based method, we used SVDquartets (Chifman & Kubatko, 2014) as implemented in PAUP* version 4.0a (build 168) with standard settings. We calculated 100 bootstrap replicates to evaluate statistical branch support. The tree was saved in the Newick format and further edited for publication in FigTree version 1.4.3 (Rambaut, 2017).

Next, we conducted a principal component analysis (PCA) of our genomic data in TASSEL version 1.5 (Bradbury et al., 2007) after removing the non‐*Amazilis* taxa from the vcf file. We first imputed missing values for the PCA using the “LD‐kNNi” method in TASSEL (see Money et al., 2015) with the following parameters: high LD Sites = 30 (default), number of nearest neighbors = 3, and the max distance between sites for LD = 100,000. We then ran the PCA and plotted the eigenvalues for PC1 versus PC2 for each individual in R v3.3.2 (R Development Core Team, 2016).

We also used the Bayesian program fastStructure (Raj et al., 2014) to examine population structuring and grouping of individuals. We first rearranged our vcf file with 34,896 SNPs and the 86 *A*. *amazilia* individuals (after removing the non‐*Amazilis* taxa) to cluster individuals at the subspecies level according to our RAxML tree (i.e., *alticola*, *azuay*, *dumerilii*, *leucophoea*, *amazilia*, and *caeruleigularis)* and then converted the vcf file into plink bed format using the program PLINK version 1.07 (Purcell et al., 2007). Next, we input the bed, bim, and fam files into fastStructure, and ran structure analyses using the available “structure.py” script. In separate model runs, we changed the number of assigned populations (K) from 2 to 6. We used a convergence criterion of 10e^−6^, and the simple prior (flat‐beta prior). We then used the “chooseK.py” script available in fastStructure, which uses a marginal likelihood estimate on multiple models runs with different K values to choose the best‐fit model for our dataset. To visualize our output meanQ files, we used the program Pophelper (Francis, 2016; available from pophelper.com) to illustrate population clustering under different values of K.

To further examine fine‐scale population structuring and clustering, we used the program fineRADstructure (Malinsky et al., 2018) to visualize estimated coancestry levels across individuals. fineRADstructure consists of several scripts to calculate coancestry between pairwise individuals (RADpainter), and subsequent clustering and population structuring between individuals (fineSTRUCTURE) using genomic SNP data. We first converted our subspecies‐organized vcf file of 34,896 SNPs to the haplotype file input format for the program using the script “hapsFromVCF” in RADpainter. Next, we calculated the coancestry matrix for all individuals using RADpainter, and then used the fineSTRUCTURE MCMC clustering algorithm with the input arguments of ×100,000, −z 100,000, and −y 1000. This MCMC algorithm repeatedly explores merging and splitting populations as well as moving individuals until a configuration is accepted that has a probability derived from the ratio of the likelihood from the previous configuration (see Lawson et al., 2012). This probabilistic process is repeated for each pair of individuals twice (each as the donor and as the recipient in the pair); hence, diagonal halves of the matrix may not be symmetrical. We then used the tree‐building algorithm in fineSTRUCTURE with the input arguments of ‐m T ‐x 10000 to create a simple tree of the individuals. Finally, we visualized the program output with the fineSTRUCTURE GUI (available from https://people.maths.bris.ac.uk/~madjl/finestructure/finestructure.html). Because the mcmc clustering and tree‐building algorithm did not provide a useful tree (i.e., did not cluster individuals of the same subspecies or geographical location together in a logical manner or in accordance with our RAxML tree), we disregarded the output tree and instead visualized the RADpainter coancestry matrix (chunks.out file) with individuals of each subspecies clustered together.

To test for potential gene flow across subspecies, we used the program Dsuite (Malinsky et al., 2021, available from github: https://github.com/millanek/Dsuite) to calculate the Patterson's D‐statistic for subspecies trios, which is the test statistic for the ABBA–BABA test (Durand et al., 2011; Green et al., 2010). The ABBA–BABA test uses the idea that phylogenetic trios can be used to test for the presence of directional introgression in non‐sister taxa. Given a phylogeny with an outgroup O, and three taxa P1, P2, and P3 with a tree topology of (O, (P3, (P2, P1))), and alleles A (ancestral) and B (derived), we would expect an equal number of (A, (B, (B, A)))s and (B, (A, (B, A)))s across all biallelic sites in the genome without introgression due to random lineage sorting. However, with introgression from P3 and P2, we would expect an abundance of the ABBA over a BABA pattern. We know that the introgression is directional from P3 to P2 since P1 still has the same ancestral allele A as the outgroup. The D‐statistic takes the ratio of the difference between ABBAs and BABAs over the total number of sites: D = (# of ABBAs − # of BABAs)/(# of ABBAs + # of BABAs). The program then tests whether the D‐statistic for each possible trio is different from 0 using a standard block jackknifing procedure (see Malinsky et al., 2021), calculates a Z‐score, and reports the associated p‐values. Dsuite takes a vcf file as input, with the option to provide an input tree. We used a vcf file containing the 34,896 SNPs of all the *A*. *amazilia* samples and the two *U*. *franciae* samples specified as an outgroup. We input our resulting RAxML tree file (see above for methods, results for RAxML tree) into the program. We report results with a jackknifing parameter of 1000 (i.e., ‐j 1000; this parameter is supposed to be larger than the extent of linkage disequilibrium (Durand et al., 2011). We tested a range of other values from 100 to 10,000, but they did not qualitatively change the results). Based on the input tree, Dsuite assessed 20 different possible subspecies trios for introgression; however, of the 20 trios, only 13 trios fall under the five possible introgression scenarios that match the geography of the subspecies (e.g., testing introgression between *azuay* and *caeruleigularis* would not make sense geographically) and where the two taxa are sister or are already the most closely related to one another in the tree (i.e., we cannot assess *azuay*‐*alticola* or *amazilia*‐*caeruleigularis* for introgression since they are already the most closely related taxa to one another in the tree) (Appendix Figure 1 in S2). We used a Bonferroni correction on the p‐values as suggested in Malinsky et al. (2021) to account for multiple hypothesis testing.

As a final test of potential gene flow between subspecies, we used the program TreeMix v.1.13 (Pickrell & Pritchard, 2012), which explores the possibility of migration events (and therefore shared ancestry) between non‐sister subspecies within the tree. First, we converted our vcf file of the 86 *A*. *amazilia* samples into the TreeMix format using the script “vcf2treemix.py” in the RAD_Tools github package (Baxter et al., 2011; available from https://github.com/CoBiG2/RAD_Tools/blob/master/vcf2treemix.py). We then ran the TreeMix program for the six subspecies, with 1000 SNP blocks (‐k 1000) to account for any linkage disequilibrium, and for the maximum of five migration events (‐m parameter). We also included the basic tree topology with no branch lengths (i.e., from our RAxML tree previously) in our program runs (‐tf parameter). We then plotted the results in R using the “plotting_funcs.R” script provided in the src folder of the TreeMix program, and reported migration edge weights for each event. The migration edge weight represents the percentage of ancestry in the second lineage that is derived from the migration event (Pickrell & Pritchard, 2012). Finally, we ran all possible 3‐population and 4‐population tests as recommended by the TreeMix authors and implemented them within the TreeMix program (see Keinan et al., 2007; Reich et al., 2009 for statistical details) to help interpret our results. In our tests, we specified a block size of 500 SNPs (‐k 500).

### 2.5 Environmental differentiation

An additional aim of our project was to compare climatic variables (i.e., temperature and precipitation) at localities of occurrence for each subspecies to gain insight into the extent to which geographic barriers (i.e., similar climatic environments across subspecies with divergence caused by geographic isolation) versus ecological expansion into new habitats (i.e., different climatic environments across subspecies) underlies the diversification of the *A*. *amazilia* subspecies. We used two different methods for this comparison using data extracted from the Worldclim2 database (Fick & Hijmans, 2017) in R using the “raster” package (Hijmans & van Etten, 2014), using a spatial resolution of 2.5 min. First, we used two variables: annual mean temperature (variable 1) and annual precipitation (variable 12) from the Worldclim2 database for each of our GPS capture locations (see Table S1), and the GPS points listed for each subspecies in Weller (2000) and Krabbe and Ridgely (2010). We only had one GPS point available for *caeruleigularis*, as the data point in Weller (2000), our sample location, and the single GPS point for this subspecies in VertNet (Constable et al., 2010; see Table S1) were effectively from the exact same location. To compare these annual mean temperature and annual precipitation variables across subspecies, we conducted Kruskal–Wallis tests (and post‐hoc pairwise Dunn tests, using the package “dunn.test” (Dinno, 2017) in R version 3.3.2 (R Development Core Team, 2016) to test if the mean temperature and annual precipitation in the habitats of each subspecies were significantly different across subspecies. For our second method, we conducted a PCA on all 19 of the Worldclim2 variables in R, and assessed whether the principal components were significantly different among subspecies using Kruskal–Wallis tests (and post‐hoc pairwise Dunn tests when necessary).

## 3 RESULTS

### 3.1 mtDNA

**FIGURE 3:**
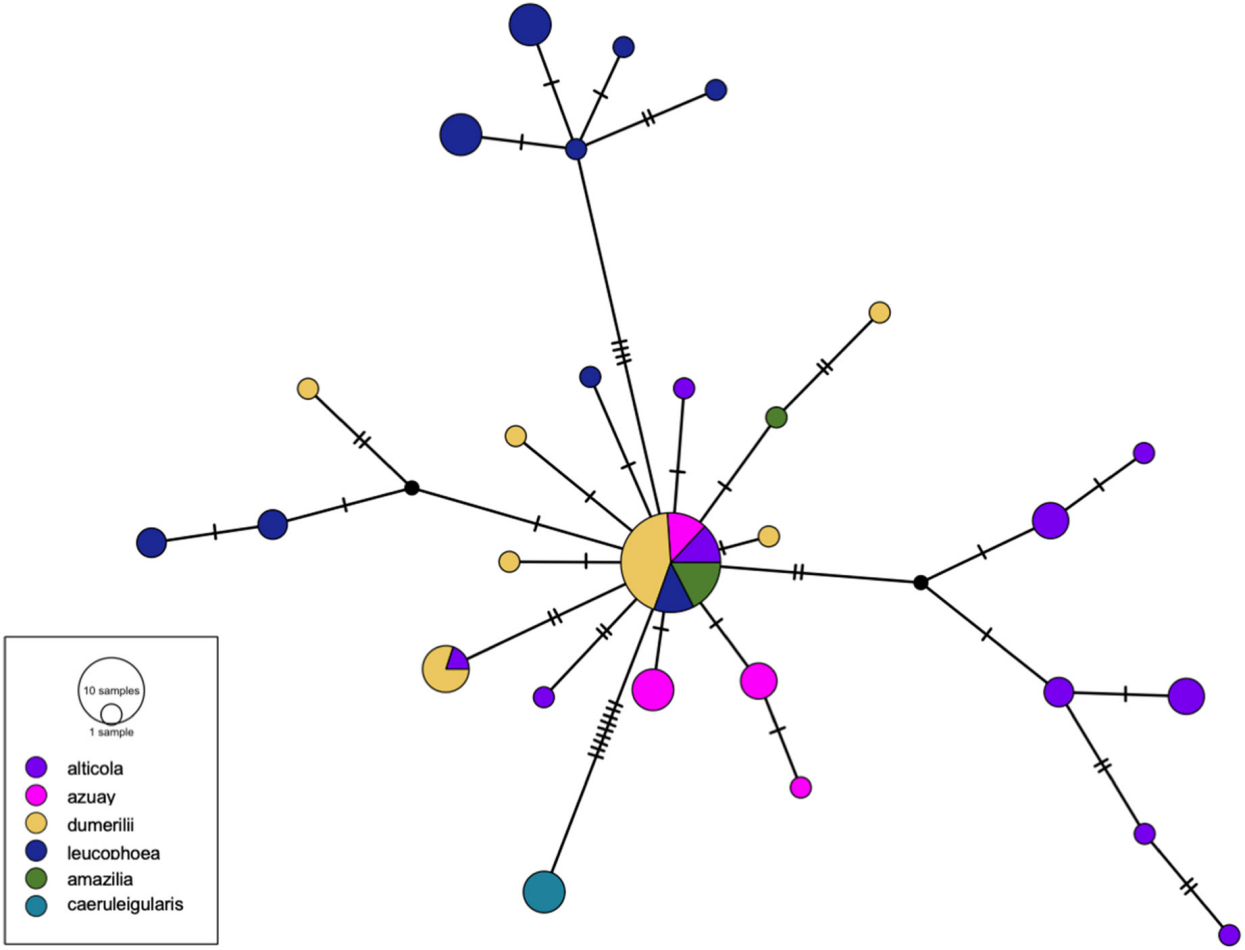
*ND2* haplotype network for *Amazilis amazilia*. The median‐spanning haplotype network for 75 individuals across subspecies is based on 1002 bp of the mtDNA gene *ND2*. The subspecies *caeruleigularis* is the only subspecies that has a distinct haplotype network. All other subspecies share a common species haplotype (23 individuals share the common haplotype across subspecies)

We sequenced and aligned 1002 base pairs of the mitochondrial gene *ND2* for a total of 75 *A*. *amazilia* individuals (17 *alticola*, 11 *azuay*, 18 *dumerilii*, 20 *leucophoea*, 5 *amazilia*, and 4 *caeruleigularis*). The median‐spanning haplotype network showed that the mtDNA haplotypes of five of the six subspecies (all but *caeruleigularis*) were intermixed and non‐monophyletic (Figure 3), and that individuals from these five subspecies shared a common mtDNA haplotype (23 individuals total, Figure 3). The only unshared mtDNA haplotype was that of *caeruleigularis*, which was separated from the common haplotype by 10 base‐pair changes, corresponding to an uncorrected sequence divergence of 1.0%. Overall, there were 10 distinct haplotypes for *alticola*, 4 for *azuay*, 7 for *dumerilii*, 9 for *leucophoea*, 2 for *amazilia*, and 1 for *caeruleigularis*, with 30 parsimony informative sites and 16 singletons in the 1002 base pair sequence.

We were also able to sequence and align six *ND2* sequences from the closely related relative *A*. *tzacatl* to the 1002 base pair *A*.* amazilia ND2* sequences. There were 101 sequence differences between the most common haplotypes of *A*. *tzacatl* and *A*. *amazilia* out of 1002 total base pairs, corresponding to an uncorrected sequence divergence of approximately 10.0%. Similarly, we were able to sequence 890 base pairs from our two samples of *U*. *franciae*. We aligned these sequences to the *A*. *amazilia* sequences, and found a total of 58 differences across the 890 base pairs of *ND2*, corresponding to an uncorrected sequence divergence of 6.51%.

The BF analysis (Log BF = 50.59) showed strong support for the relaxed log‐normal clock model over the strict clock model. The birth‐death prior rendered a better performance than the Yule prior and, for the same reason, we chose HKY + G as the site model instead of the GTR + G model (all ESS > 200). We decided to retain the HKY + G, instead of the GTR +G model chosen by PartitionFinder, to avoid over parametrization of the model. Our maximum clade credibility tree based on the 0.029 s/s/million years rate (Lerner et al., 2011; Appendix Figure 2 in S2), estimated an age of 0.211 million years (95% Highest Posterior Probability Density [HPD] = 0.111–0.325 million years) for *A*. *amazilia* and of 0.014 million years (95% Highest Posterior Probability Density [HPD] = 0.0009–0.0340 million years) for *caeruleigularis*. The ages of other subspecies within *A*. *amazilia* were impossible to estimate because these taxa were not monophyletic. On the other hand, *U*. *franciae* and *A*. *amazilia* last shared a common ancestor about 2.092 million years ago (95% Highest Posterior Probability Density [HPD] = 1.533–2.720 million years), and *A*. *amazilia* and *A*. *tzacatl* last shared a common ancestor about 2.753 million years ago (95% HPD = 1.839–3.657 million years), (see Appendix Table 3 in S2 for divergence estimates using 0.0125 and 0.0068 substitutions/site/million years).

### 3.2 Phylogeny

**FIGURE 4:**
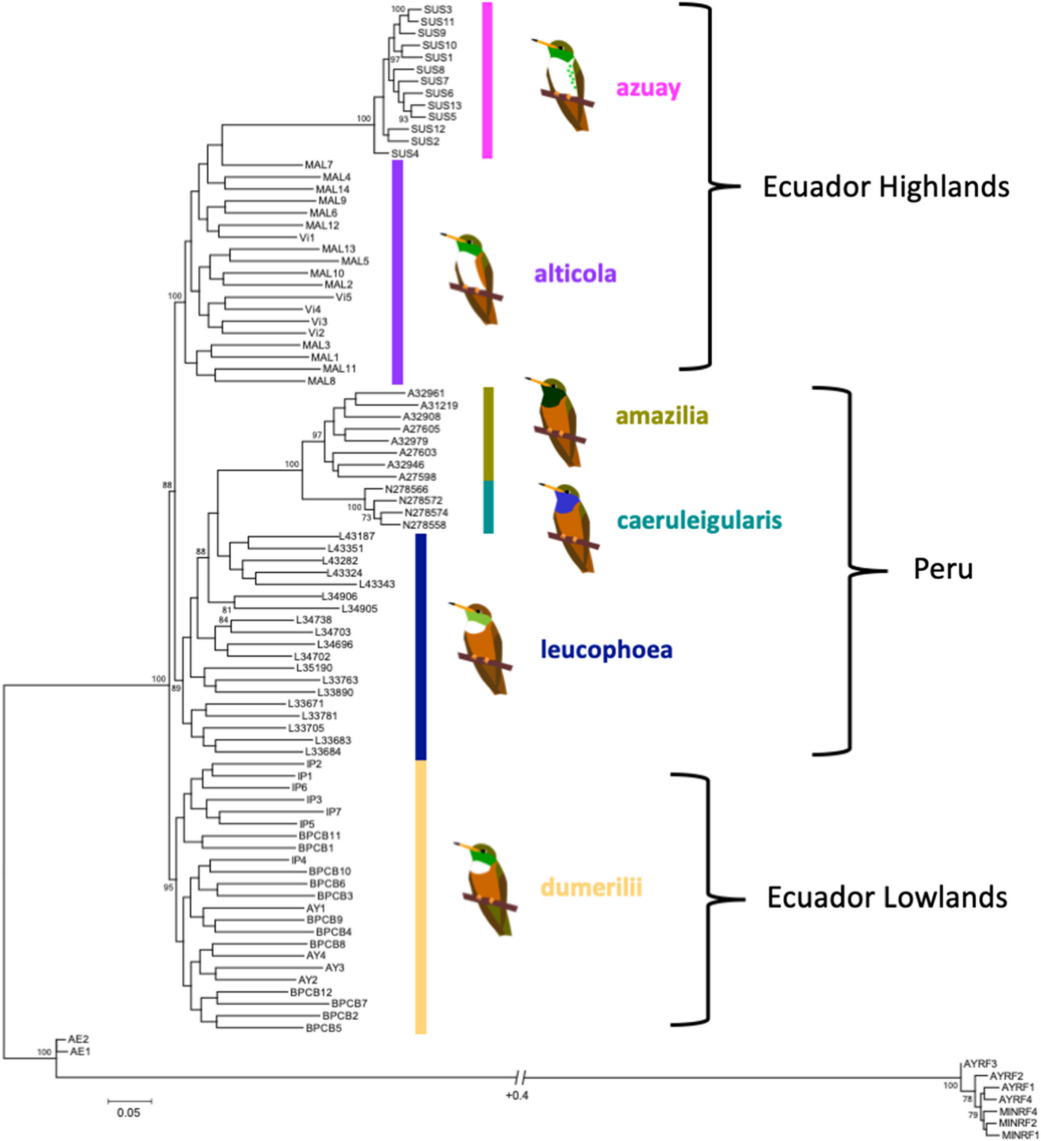
RAxML phylogenetic tree of the *Amazilis amazilia* subspecies complex. Our maximum likelihood‐based tree from the program RAxML is based on our dataset of 34,896 SNPs for all six subspecies of *A*. *amazilia* (86 individuals total) and 9 samples from closely related relatives (2 *Uranomitra franciae* and 7 *Amazilia tzacatl*). Only bootstrap percentages ≥70 are shown on the tree

Our RAxML maximum‐likelihood phylogeny was based on a matrix of 34,896 SNPs from 86 *A*. *amazilia* and 9 closely related DNA samples (7 *A*. *tzacatl* and 2 *U*. *franciae*), and had 31.49% gaps (percentage of missing data). The phylogeny showed a clear separation of the six subspecies (Figure 4). The samples fell into three distinct clades with high bootstrap support: a highlands Ecuador clade (subspecies *azuay* and *alticola*; 100% bootstrap support), a Peru clade (subspecies *leucophoea*, *amazilia*, and *caeruleigularis*, 89% bootstrap support), and a lowlands Ecuador clade (subspecies *dumerilii*, 95% bootstrap support). The lowlands Ecuador clade is sister to the highlands Ecuador and Peru clades. Within the highlands Ecuador clade, the subspecies *azuay* appears to have arisen from the secondary isolation of individuals from the *alticola* group (100% bootstrap support). Similarly, within the Peru clade, the sister taxa of *amazilia* (97% bootstrap support) and *caeruleigularis* (100% bootstrap support) appear to have arisen from the *leucophoea* lineage.

In contrast, in our coalescent‐based SVDquartets tree (Appendix Figure 3 in S2), the highlands Ecuador subspecies are divided into two separate clades. The subspecies *azuay* (100% bootstrap support) is the earliest diverging of all *A*. *amazilia* subspecies, followed by *alticola* (70% bootstrap support). Subspecies *dumerilii* is next to diverge (96% bootstrap support), followed by the Peruvian clade (69% bootstrap support). Similar to the maximum likelihood phylogeny within the Peruvian clade, the clade with subspecies *amazilia* (100% bootstrap support) and *caeruleigularis* (100% bootstrap support) is derived from the *leucophoea* lineage (paraphyletic). Overall, both phylogenies show distinct geographical subspecies groupings.

### 3.3 Population structuring

**FIGURE 5:**
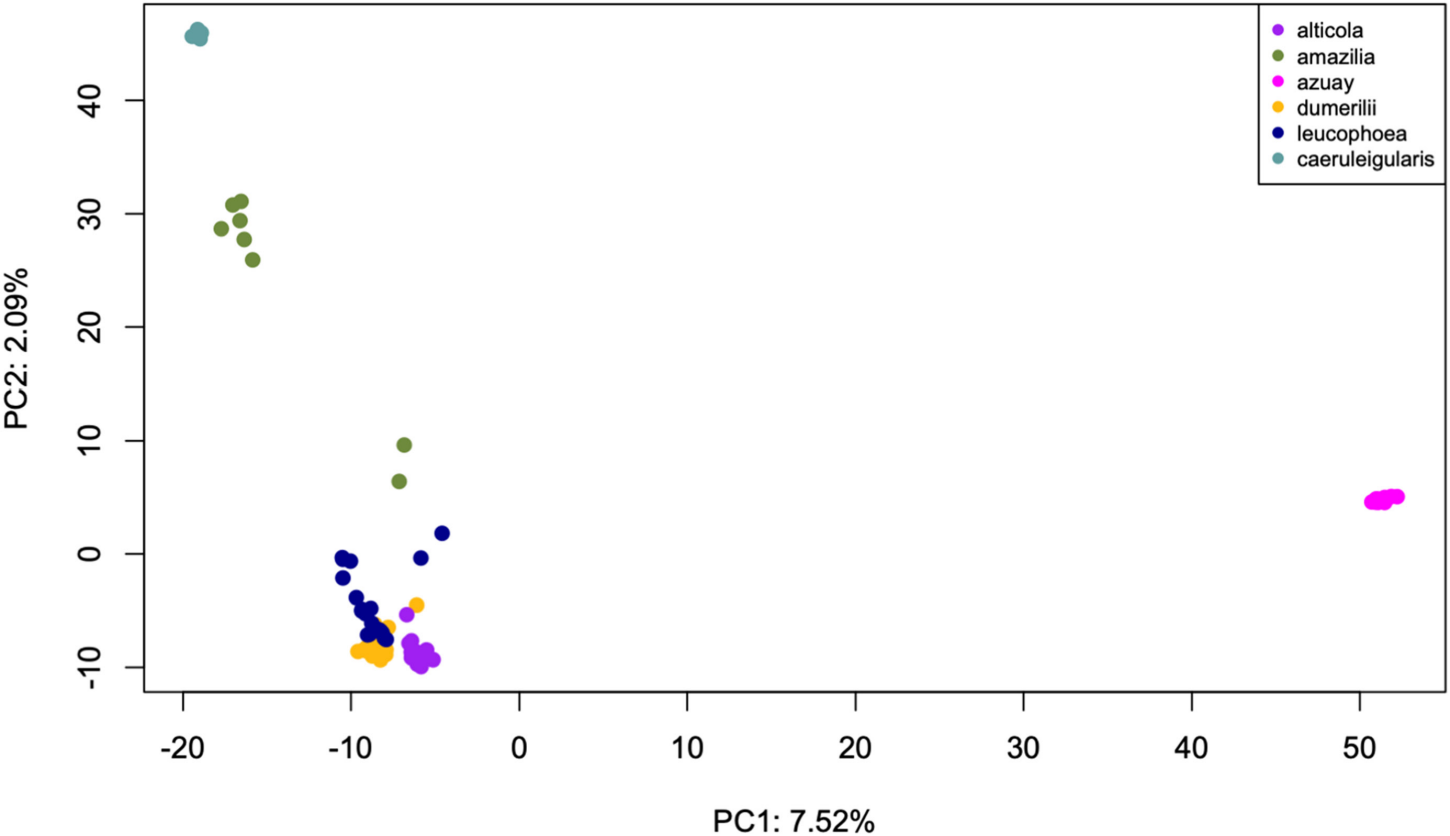
PCA of genomic data. We conducted a PCA on the genomic dataset of 34,896 SNPs in TASSEL using the “LD‐kNNi” impute method for missing data (see text for details). PC1 separates *azuay* from the other five subspecies, whereas PC2 separates *caeruleigularis* and most *amazilia* individuals from a cluster of *alticola*, *dumerilii*, and *leucophoea*. The two *amazilia* subspecies samples (MSB:BIRD:27603 and MSB:BIRD31219) that are more genetically similar to the cluster of *dumerilii*, *leucophoea*, and *alticola* individuals were collected in Lima, Peru, same as all other *amazilia* subspecies samples (see Table S1)

The PCA in TASSEL indicated that *azuay* is the most genetically distinct subspecies, as the first principal component explained 7.52% of the genomic differentiation and clearly separated *azuay* from the other five subspecies (Figure 5). The second principal component explained 2.09% of the genomic variation, and separated *caeruleigularis* and *amazilia* from *alticola*, *dumerilii*, and *leucophoea* (Figure 5). However, individuals of *alticola*, *dumerilii*, and *leucophoea* do show some overlap in both PC1 and PC2 (Figure 5), suggesting they are genetically similar and may experience contemporary gene flow or have had recent gene flow.

**FIGURE 6:**
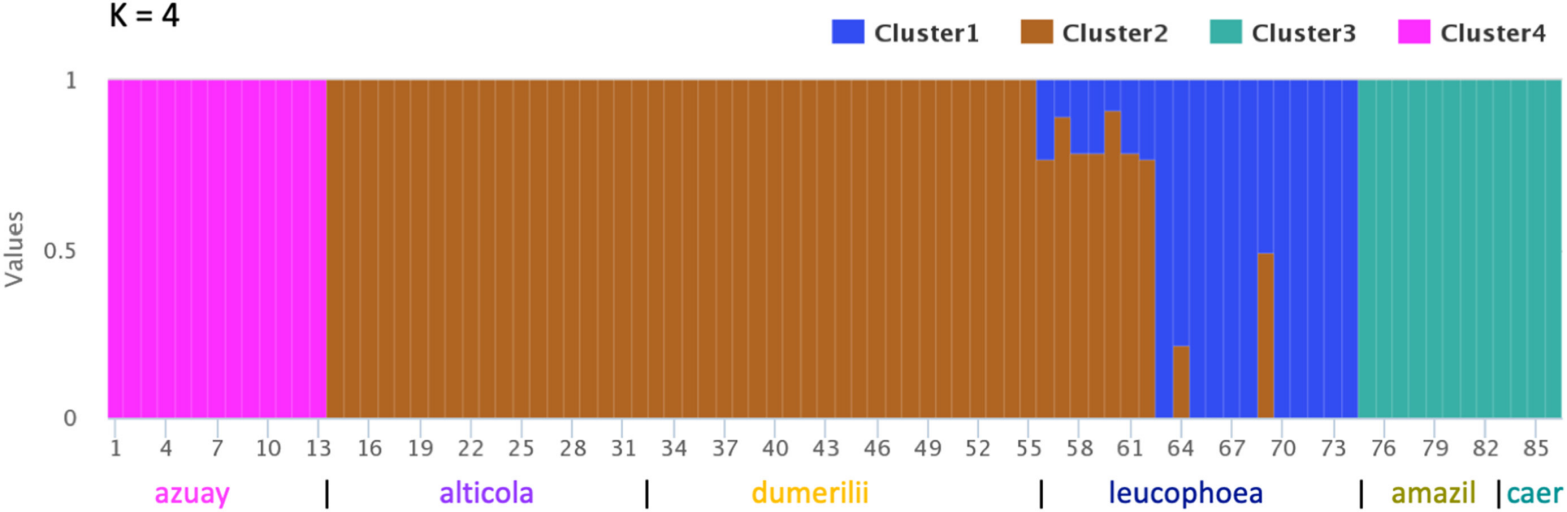
fastStructure plot for K = 4. K = 4 was the best‐fit model using the choose.py script for K ≥ 4. Different colors on plots represent different assigned genetic clusters

Using the program fastStructure, we found that individuals of *A*. *amazilia* were clustered into distinct genetic groups based on subspecies. K = 4 was the best‐fit model for explaining the structure in the data using the fastStructure script “choose.py” for runs K ≥ 4. With K = 4, fastStructure found four distinct genetic clusters: (1) *azuay*, (2) *amazilia‐caeruleigularis*, and (3) *alticola‐dumerilii*, with (4) *leucophoea* as the final genetic cluster (Figure 6). Nine of the *leucophoea* individuals show genetic admixture between the *alticola‐dumerilii* and *leucophoea* clusters (see Appendix Figure 4 in S2 for other models results based on K = 2, 3, 5, and 6).

We used the fineRADstructure program to calculate pairwise coancestry values between all *A*. *amazilia* individuals. We found that the program showed higher coancestry values and structuring for both *azuay* and *caeruleigularis*, but otherwise detected little to no population structuring across the other four subspecies (*dumerilii*, *alticola*, *leucophoea*, and *amazilia*; Appendix Figure 5 in S2).

### 3.4 Introgression in non‐sister subspecies

We used the program Dsuite to calculate D‐statistics for possible introgression between non‐sister subspecies trios. Over our five possible scenarios of introgression between non‐sister subspecies (based on the phylogenetic tree and geography, see Appendix Figure 1 in S2), we found support for introgression in almost all cases (Appendix Table 4 in S2): *dumerilii* to *alticola* (3 of 4 trios), *dumerilii* to *azuay* (2 of 2 trios), *dumerilii* to *leucophoea* (2 of 3 trios), *alticola* to *leucophoea* (2 of 2 trios), and *leucophoea* to *amazilia* (1 of 1 trio). The only trios that were not significant were trios in which *azuay* was specified as P1 in the trio.

We also used the program TreeMix to test for potential historical gene flow across subspecies. Using all five possible migration events specified in TreeMix, we found that the program indicated these top five patterns of migration (in order of strength from strongest to weakest): (1) *alticola* to *leucophoea* (migration edge weight 0.31), (2) *leucophoea* to *amazilia* (migration edge weight 0.27), (3) *azuay* to *amazilia* (migration edge weight 0.07), (4) *caeruleigularis* to *alticola* (migration edge weight 0.02), and (5) *caeruleigularis* to *dumerilii* (migration edge weight 0.01) (Appendix Figure 6 in S2). However, based on current geography and the strength of migration weight estimates, the only likely migration patterns specified by TreeMix are patterns 1 and 2 (Appendix Figure 1 in S2: migration pattern 1 matches up with scenario 4, and migration pattern 2 matches up with scenario 5), given *azuay* and *amazilia*, *caeruleigularis* and *alticola*, and *caeruleigularis* and *dumerilii*, respectively, are geographically distant from one another. Therefore, we only report the 3‐population and 4‐population statistical tests supporting the first two TreeMix migration patterns (i.e., 1) *alticola* to *leucophoea* and 2) *leucophoea* to *amazilia*).

Using 3‐population tests implemented within the program TreeMix, we found support for migration pattern 2 from the subspecies *leucophoea* to *amazilia*, as one of four 3‐population tests with *amazilia* specified as the admixed population and with *leucophoea* included (i.e., [*amazilia*: *leucophoea*, x], where x = another subspecies) indicated admixture (f_3_ = −0.0001, Z = −5.2, *p* < .001). We did not find support for migration pattern 1, as all four tests with *alticola* specified as the admixed population with *leucophoea* included (i.e., [*alticola*: *leucophoea*, x], where x = another subspecies) did not indicate admixture (f_3_ ≥ 0.00007; positive f_3_ values indicate no admixture). With 4‐population tests, the aim was to find 4‐population trees that match the real‐tree topology but that fail the 4‐population test (i.e., have a |Z‐score| >2), which would suggest the presence of gene flow across the true tree. We found support for migration pattern (1) *alticola* to *leucophoea* in three trees (f_4_ ≥ 0.00007, Z ≥ 5.1, *p* < .001 for all three trees), and support for migration pattern (2) *leucophoea* to *amazilia* in one tree (f_4_ = 0.0001, Z = 6.4, *p* < .001). Therefore, 3‐population tests supported migration pattern 2 from *leucophoea* to *amazilia*, whereas 4‐population tests supported migration from both migration pattern 1: *alticola* to *leucophoea* and migration pattern 2: *leucophoea* to *amazilia*.

### 3.5 Habitat differentiation

To examine whether environmental differences exist in the habitats of each subspecies, we assessed whether annual mean temperature and annual precipitation were significantly different across subspecies (given we only have one GPS point for *caeruleigularis*, all comparisons with *caeruleigualris* were non‐significant). The average annual mean temperature (±SE) in ºC for all subspecies were as follows: *alticola* (*n* = 7) 18.1 ± 1.3, *amazilia* (*n* = 12) 18.8 ± 0.2, *azuay* (*n* = 3) 15.9 ± 1.5, *caeruleigularis* (*n* = 1) 20.7 ± NA, *dumerilii* (*n* = 28) 24.3 ± 0.27, and *leucophoea* (*n* = 28) 19.2 ± 0.67. Using a Kruskal–Wallis test, the temperature was significantly different across subspecies (Kruskal–Wallis, X^2^ = 45.8, *df* = 5, *p* < .001). Using post‐hoc pairwise Dunn tests with a Bonferroni correction, *dumerilii* was significantly different from all other subspecies (except *caeruleigularis*) in annual mean temperature (Appendix Table 5 in S2).

The average annual precipitation (±SE) in mm for all subspecies was as follows: *alticola* (*n* = 7) 956.1 ± 109.9, *amazilia* (*n* = 12) 17.4 ± 5.9, *azuay* (*n* = 3) 616.7 ± 15.4, *caeruleigularis* (*n* = 1) 5 ± NA, *dumerilii* (*n* = 28) 899.1 ± 97.8, and *leucophoea* (*n* = 28) 217.0 ± 50.2. Using a Kruskal–Wallis test, annual precipitation was significantly different across subspecies (Kruskal–Wallis, X^2^ = 52.6, *df* = 5, *p* < .001). Using post‐hoc pairwise Dunn tests with a Bonferroni correction, there were significant differences between *alticola*‐*amazilia*, *alticola*‐*leucophoea*, *dumerilii*‐*amazilia*, and *dumerilii*‐*leucophoea* in annual precipitation (Appendix Table 5 in S2). To further visualize the differences in annual mean temperature and precipitation across subspecies, we plotted annual mean temperature versus annual precipitation for each subspecies’ GPS points (Appendix Figure 7 in S2, 80% CI ellipses for each subspecies drawn for visualization).

**FIGURE 7:**
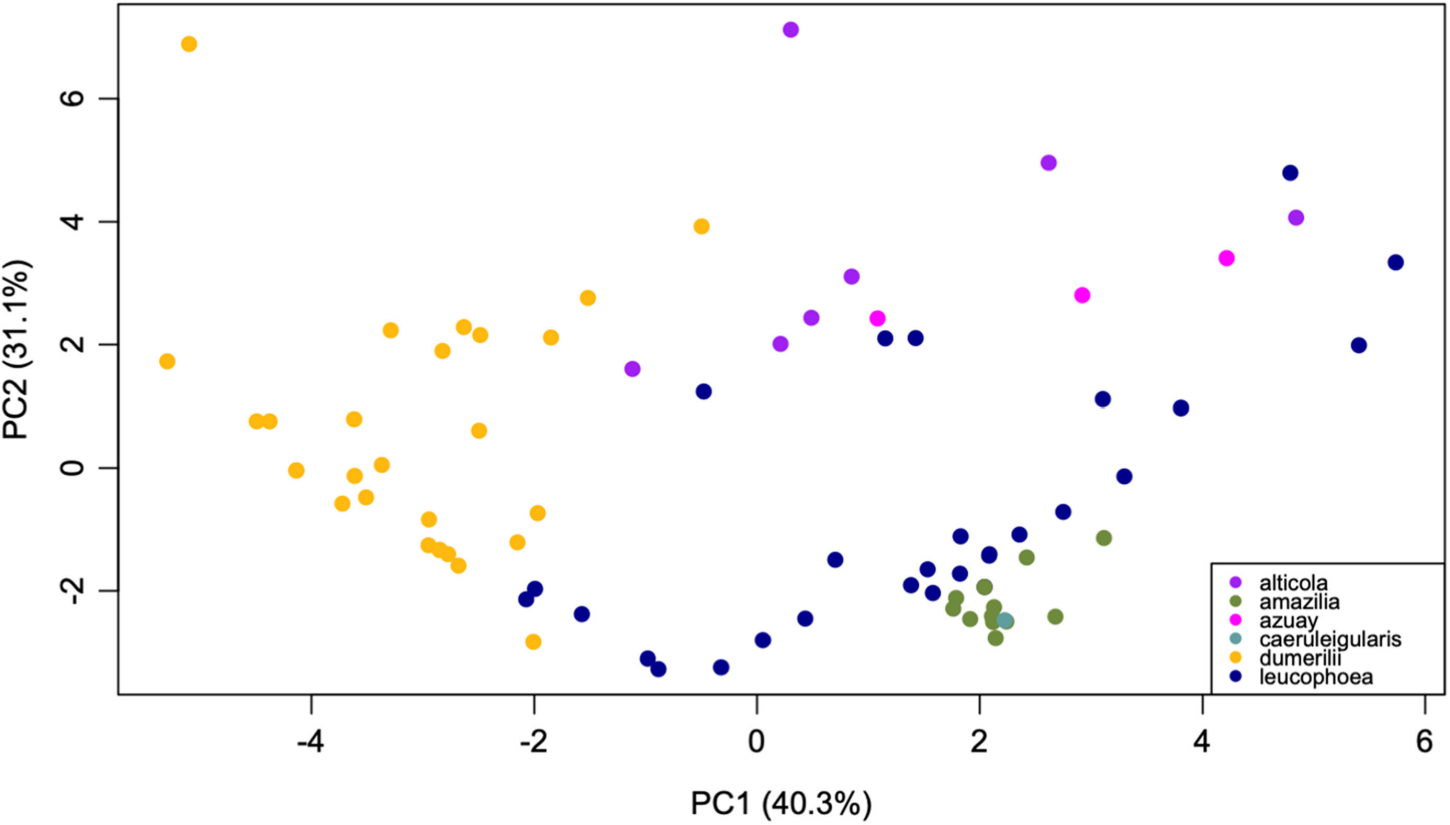
PC1 vs. PC2 for all 19 Worldclim2 variables. Both PC1 and PC2 have significantly different subspecies groupings (PC1 differentiates *dumerilii* from the other subspecies and PC2 differentiates *alticola‐azuay* from *amazilia‐leucophoea* (see text for details)

For our second method of examining environmental differences across subspecies, we conducted a PCA on all 19 of the Worldclim2 variables from each GPS point and compared these values across subspecies. Overall, we found that the PCA was able to distinguish between subspecies using environmental variables (Figure 7). We report only the values of the first five principal components (PCs), as they cumulatively explain 93.4% of the variance across subspecies. PC1 explained 40.3% of the variation, and was positively associated with mean diurnal range, temperature seasonality, temperature annual range, and precipitation in the driest month, and negatively associated with all other Worldclim2 variables (Appendix Table 6 in S2). PC1 was significantly different across subspecies (Kruskal–Wallis test, X^2^ = 53.9, *df* = 5, *p* < .001). Using post‐hoc pairwise Dunn tests with a Bonferroni correction, PC1 was significantly different between *dumerilii*‐*alticola* (*Z* = 3.4, *p* = .005), *dumerilii*‐*amazilia* (*Z* = 5.71, *p* < .001), *dumerilii*‐*azuay* (*Z* = 3.44, *p* = .004), and *dumerilii*‐*leucophoea* (*Z* = −5.88, *p* < .001) (all other pairwise comparisons |Z| < 2.06, *p* > .3). Therefore, PC1 can be interpreted as distinguishing *dumerilii* from all other subspecies (Figure 7).

PC2 explained 31.1% of the variation, and was positively associated with mean diurnal range, isothermality, annual precipitation, precipitation in the wettest month, precipitation in the driest month, precipitation in the wettest quarter, precipitation in the driest quarter, precipitation in the warmest quarter, and precipitation in the coldest quarter, and was negatively associated with all other Worldclim2 variables (Appendix Table 6 in S2). PC2 was significantly different across subspecies (Kruskal–Wallis test, X^2^ = 37.7, *df* = 5, *p* < .001). Using post‐hoc pairwise Dunn tests with a Bonferroni correction, PC2 was significantly different between *alticola*‐*amazilia* (*Z* = 4.91, *p* < .001), *alticola*‐*leucophoea* (*Z* = 3.76, *p* = .001), *amazilia*‐*azuay* (*Z* = −3.65, *p* = .002), *amazilia*‐*dumerilii* (*Z* = −4.04, *p* = .004), and very close to significant between *azuay*‐*leucophoea* (Z = 2.66, *p* = .06) (all other pairwise comparisons |*Z*| < 2.46, *p* > .10). Therefore, PC2 can be seen as distinguishing *alticola‐azuay* from *amazilia‐leucophoea*, as well as additional differentiation between *amazilia* and *dumerilii* (Figure 7).

PC3 explained 11.2% of the variation, and was positively associated with annual mean temperature, mean diurnal range, isothermality, max temperature warmest month, temperature annual range, mean temperature wettest quarter, mean temperature coldest quarter, annual precipitation, precipitation wettest month, precipitation seasonality, precipitation wettest quarter, and precipitation warmest quarter, and was negatively associated with all other Worldclim2 variables (Appendix Table 6 in S2). PC3 was significantly different across subspecies (Kruskal–Wallis test, X^2^ = 17.0, *df* = 5, *p* = .004). Using post‐hoc pairwise Dunn tests with a Bonferroni correction, PC3 was significantly different between *amazilia*‐*leucophoea* (*Z* = −3.39, *p* = .005). Although PC4 explained 5.8% of the variation and was significantly different across subspecies (Kruskal–Wallis test, X^2^ = 13.1, *df* = 5, *p* = .02), no subspecies comparisons were significant using the post‐hoc pairwise Dunn tests with a Bonferroni correction (all comparisons *p* > 0.13). Similarly. PC5 explained 4.9% of the variation, but was not significantly different across subspecies (Kruskal–Wallis test, X^2^ = 6.4, *df* = 5, *p* = .27).

## 4 DISCUSSION

### 4.1 Phylogeny

We found that our phylogenies of *A*. *amazilia* based on the genomic dataset of 34,896 SNPs match the current classification of the six subspecies. The six defined subspecies were clearly separated from each other in both phylogenies, as all individuals within each subspecies clustered together, and no individuals were mismatched across subspecies. In addition, the subspecies fell into distinct, highly supported clades associated with geography with high bootstrap support: a highlands Ecuador clade (*alticola* and *azuay*; however, split into two separate clades in the SVDquartets tree), a lowlands Ecuador clade (*dumerilii*), and a Peruvian clade (*leucophoea*, *amazilia*, and *caeruleigularis*). Although it has often been questioned whether the subspecies *alticola* (the Loja Hummingbird) should be considered a full species (Krabbe & Ridgely, 2010; Weller, 2000), our phylogenies suggest that *alticola* should remain a subspecies, due to its genetic similarity with *dumerilii* and *leucophoea* (also see gene flow section below). In contrast, *azuay* is by far the most genetically distinct subspecies, and could be considered at the level of a full species, due to its geographical isolation, distinct plumage (see Krabbe & Ridgely, 2010), genetic distinctiveness as shown by our genomic analyses (see below), and potential early divergence from the rest of the subspecies (as supported by our SVDquartets phylogeny).

Weller (2000) predicted *dumerilii* and *leucophoea* formed a clade due to phenotypic similarity, and that south to north expansion of the subspecies was likely based on phenotypic characters. However, our phylogenies suggest that north to south expansion of the species was more likely. In addition, *leucophoea* is more closely related to *amazilia* and *caeruleigularis* than to *dumerilii*. This result is interesting because as Weller (2000) stated, both *leucophoea* and *dumerilii* have a prominent white throat patch and red underbelly, and are larger in size. However, the phenotypic similarity between *leucophoea*, *dumerilii*, and *alticola* may stem from gene flow across these three subspecies (see below), or could also be a result of shared ancestral characters.

### 4.2 Gene flow and population structuring across subspecies

We used five different genomic analyses designed to assess genetic differentiation, gene flow, and population structuring across the six subspecies of *A*. *amazilia*. Taking all methods into account, our results provide strong evidence of genetic similarity and gene flow across the three subspecies of *dumerilii*, *leucophoea*, and *alticola* (Appendix Figure 1 in S2), with support for introgression or strong genetic similarity from at least 4 of the 6 analyses for each pair. These three subspecies have the potential for large areas of contact across the southern regions of Ecuador and the Ecuadorian‐Peruvian border (Schulenberg et al., 2010; Weller et al., 2019), which could facilitate gene flow across areas of contact. In addition, the PCA supported genetic similarity, as there was a clear overlap in the genomic PCA of these three subspecies. fastStructure also supported genetic similarity, and in scenarios K ≥ 4, two of these three subspecies (either *alticola*‐*dumerilii*, or *alticola*‐*leucophea*) were grouped together into one genetic population. Phenotypically, the three subspecies appear most similar to each other, with each of the three possessing a prominent white throat patch, a rufous underbelly, and a green or rufous backside (mantle and rump) and tail coverts. Phenotypic intergradation is visible between *alticola* and *dumerilii* in Ecuador, particularly in tail covert color and the amount of underside rufous belly coloration (Weller et al., 2019; S. Cowles pers. obs.).

In contrast, it appears that the subspecies *azuay* is the most genetically isolated subspecies, as the first principal component clearly separated *azuay* from the rest of the subspecies in the genomic PCA, and it was the most genetically differentiated from other subspecies in fastStructure for *K* = 2. The only method that supported any gene flow between *azuay* and another subspecies was D‐statistics in Dsuite; however, Dsuite found gene flow in all subspecies pairwise scenarios, suggesting that it is unable to discern subspecies differentiation across this recently diverged taxonomic group. Phenotypically, *azuay* is the only subspecies with little to no rufous coloration on the chest and belly and an incomplete gorget (Krabbe & Ridgely, 2010; S. Cowles pers. obs.). *Caeruleigularis* and *amazilia* were the next most genetically isolated subspecies, based on the second principal component in the genomic PCA and the fastStructure results for *K* = 3. The subspecies *amazilia* and *caeruleigularis* are the only subspecies to lack both a white throat patch and white belly coloration (as the belly is entirely rufous), and have distinctly colored gorgets (dark green and bluish‐purple, respectively) compared to the medium green of the other four subspecies (Weller, 2000). In addition, our results show genetic similarity between *leucophoea* and *amazilia* in Peru (Scenario 5 from Appendix Figure 1 in S2), particularly supported by our PCA and fastStructure results. Together with the TreeMix results, our findings suggest introgression between *leucophoea* and *amazilia* has likely taken place.

Although our various genomic methods gave slightly conflicting results, the consilience of evidence suggested gene flow among *alticola*, *dumerilii*, and *leucophoea*, some gene flow between *leucophoea* and *amazilia*, and strong genetic isolation of *azuay* and *caeruleigularis*. We suggest that it is imperative to use multiple complementary genomic analyses to obtain a robust inference of population structuring and gene flow among subtlety‐differentiated lineages (e.g., Beckman et al., 2018). Some methods are less able to detect structuring and/or gene flow in recently diverged taxa like subspecies and may have a better potential for assessing species‐level relationships. For example, the fineRADstructure clustering algorithm may work better for more highly differentiated populations at the species level, as it was unable to detect much population structure beyond the two most genetically differentiated subspecies *azuay* and *caeruleigularis*. Similarly, the Dsuite algorithm may work better for testing introgression at the species level, as it detected introgression in all five introgression scenarios tested (*alticola*‐*dumerilii*, *azuay*‐*dumerilii*, *dumerilii*‐*leucophoea*, *alticola*‐*leucophoea*, and *amazilia*‐*leucophoea*). The only trios that were not significant were trios in which *azuay* was specified as P1 in the trio. This may be due to the fact that Dsuite and the calculation of D‐statistics only take into account tree topology and not the level of genetic divergence. We know that *azuay* is the most genetically distinct subspecies from the genomic PCA and faststructure results, and not taking this into account likely affected the calculation of the D‐statistics.

Even though there was support for introgression from *dumerilii* to *azuay* (scenario 2 in Appendix Figure 1 in S2) using D‐statistics, the genetic clusters shown by the phylogenetic trees, fastStructure, and PCA results suggested otherwise. In both of the two supported *azuay*‐*dumerilii* trios, P1 was either *amazilia* or *caeruleigularis*, which means the three subspecies used in the trio were not particularly closely related to one another. There was a signature of latitudinal differentiation in the genomic structure of the subspecies (PC2 in the genomic PCA in Figure 5 separates subspecies north to south across their ranges, see Figure 2). Therefore, it would make sense that D‐statistics would predict that a mid‐latitude subspecies (e.g., *dumerilii*) shows introgression with a northern subspecies (e.g., *azuay*) compared to a southern subspecies (e.g., *caeruleigularis*). This prediction of introgression happens specifically in cases where the most southern subspecies (*caeruleigularis* or *amazilia*) is specified to be more closely related to the northern subspecies (*azuay*) based on the trio topology. Therefore, we suggest that *alticola*, *dumerilii*, and *leucophoea* have a history of introgression, as do *leucophoea* and *amazilia*, but that the *azuay* and *dumerilii* subspecies have not introgressed.

The dearth of well‐supported within‐subspecies clustering, especially in our *dumerilii* samples from Ecuador, also suggested ample geographic dispersal. We collected *dumerilii* samples from field sites over 200 km apart, and even separated by a water barrier (Ayampe, Guayaquil, and Isla Puná), yet did not recover strong bootstrap support (≥70) for the monophyly of these geographic groups in our phylogenetic trees. This result suggests that dispersal is likely to facilitate gene flow in the absence of substantial geographic barriers.

### 4.3 mtDNA patterns and timing of divergence

Our *ND2* haplotype network shows that five of the six subspecies share a common haplotype for this gene (all but *caeruleigularis*), which is in contrast to the phylogenies based on genomic data that showed a clear separation of subspecies. Most surprisingly, the majority of *azuay* individuals shared this common species haplotype, even though the genomic data from nuclear SNPs placed *azuay* as the most genetically isolated subspecies. *Caeruleigularis* was the only subspecies with a differentiated haplotype for *ND2*, and we estimate it to have diverged from the other subspecies extremely recently (14,000 years ago based on the 0.029 s/s/million years rate). This unresolved mtDNA network is likely due to incipient diversification with partial sorting, and suggests that the diversification of the *A*. *amazilia* subspecies was relatively recent and rapid. We think that the distinct mtDNA haplotype of *caeruleigularis* may result from its possible evolutionary history of an extreme bottleneck and peripheral isolation in the Nazca Valley of Peru (see below). Our estimates of divergence times between *A*. *amazilia* and its closely related relatives *U*. *franciae* (2.09 million years) and *A*. *tzacatl* (2.75 million years) are in line with McGuire et al. (2014)’s estimates that most of the *Amazilia* genus evolved in the late Miocene and Pliocene (11.63–2.58 million years ago).

In birds, it is common to find mismatches between histories based on mtDNA genes and genomic‐wide data within phylogeographic studies (so called mito‐nuclear discordance, see Funk and Omland (2003), McKay and Zink (2010), Toews and Brelsford (2012), and Hill (2017) for comprehensive overviews), and this scenario can be indicative of isolation coupled with periods of gene flow (e.g., Block et al., 2015; Hogner et al., 2012; Webb et al., 2011; Zarza et al., 2016), selection on specific mtDNA genes (e.g., Morales et al., 2015), differential introgression of mtDNA versus nuclear DNA (e.g., Carling & Brumfield, 2008; Sardell & Uy, 2016), or incomplete lineage sorting of mtDNA haplotypes (e.g., Harvey & Brumfield, 2015). In the closely related species *A*. *tzacatl* that ranges from Southeastern Mexico down to the central coast of Ecuador, Miller et al. (2011) found five distinct mtDNA clades with divergence up to 2.4%; however, these clades did not fully correspond with the five currently recognized subspecies of *A*. *tzacatl*. In *A*. *amazilia*, we found that although genomic data showed that the six subspecies are genomically distinct from one another, the mtDNA haplotypes for each subspecies were only distinct for *caeruleigularis*. This pattern suggests that assessing divergence histories based on mtDNA alone, particularly within the case of closely related taxa such as subspecies, may provide insufficient phylogenetic resolution. Genome‐wide SNP datasets in concert with mtDNA, as used in the present study, can reveal diversification dynamics that occurred too rapidly to leave a signature in mtDNA (also see Funk et al., 2021).

### 4.4 Expansion followed by geographic isolation

According to our two analyses assessing temperature and precipitation variables, the six subspecies of *A*. *amazilia* inhabit three fairly distinct environmental combinations: hotter and wetter (subspecies *dumerilii*, tropical dry forest in the Ecuador lowlands), cooler and wetter (subspecies *azuay* and *alticola*, subtropical forest in the Ecuador Highlands), and drier and intermediate in temperature (subspecies *leucophoea*, *amazilia*, and *caeruleigularis*, desert scrub and dry coastal environments on the Peruvian coast). These distinct environmental combinations suggest that ecological expansion into new habitats between the Ecuador lowlands, the Ecuador highlands, and the desert environments of Peru was an important driver of subspecies diversification in *A*. *amazilia*. Subsequently, the isolation of *azuay* in the Yunguilla Valley of Ecuador, and *amazilia* and *caeruleigularis* from *leucophoea* in the southern coastal regions and Nazca/Ica regions of Peru likely drove additional subspecies differentiation. We did not find any significant differences in environmental characteristics between *alticola* and *azuay*, and very few differences between *leucophoea*, *amazilia*, and *caeruleigularis* (only PC3 was different between *leucophoea* and *amazilia* in the PCA of the 19 Worldclim variables; average temperature and precipitation were not significantly different between the two), supporting at least a partial role for geographical isolation in driving the divergence of *azuay* and *alticola*, and *amazilia* and *caeruleigularis* from *leucophoea*. Our results suggest that a combination of both ecological expansion into new habitats and geographical isolation were critical drivers of subspecies diversification in *A*. *amazilia*. These results are consistent with Graham et al. (2009)’s broader study of hummingbird communities, which found that temperature, precipitation, and vegetation structure were important factors in structuring hummingbird community composition, and with Rodríguez‐Gómez et al. (2021)’s study on the Azure‐crowned Hummingbird (previously in the *Amazilia* genus, now *Saucerottia cyanocephala*), which found that both isolation and habitat differences played an important role in subspecies diversification.

The subspecies *azuay* is found only within the Yunguilla Valley (Río Jubones drainage system) of the Azuay province of Ecuador, which is approximately a 100‐mile dry valley that extends from the south of Cuenca to Machala along the coast. This valley is isolated by more humid forests on the western slope of the western Andes, the cordilleras of Chilla‐Tioloma and Cordoncillo, and the western slope of the eastern Andes (Krabbe & Ridgely, 2010). Another bird species, the Pale‐headed Brush Finch (*Atlapetes pallidiceps*) is endemic to the Yunguilla Valley (Krabbe & Ridgely, 2010), which suggests that this valley could be an isolated refuge for some time and allowed for the divergence of the *azuay* subspecies.

Similarly, the most southern subspecies *caeruleigularis* is also found within a well‐isolated valley: the small Ica‐Nazca region of Peru. This region is surrounded by dry desert plains; however, the large Pasco and Ica Rivers in the Río Grande de Nazca drainage have provided a water source in the dry desert conditions since ancient times. Within the Ica and Nazca River Valleys, other rare Peruvian endemics, such as the plant *Prosopis limensis* and *Onoseris humboldtiana*, are found (Whaley et al., 2019). Interestingly, the Nazca Valley has been home to one of the most ancient human civilizations due to its water source. The Nazca people developed subterranean irrigation systems (Schreiber & Lancho Rojas, 1995), and this human‐induced agriculture in the Nazca Valley might have had positive impact on *caeruleigularis* through the increase in food availability.

Finally, we note that the most geographically isolated populations of *azuay* and *caeruleigularis* are the most genetically distinct subspecies. This could be due to genetic bottlenecks that occurred during the initial isolation of a few individuals in the formation of these subspecies, which allowed for genetic drift and small population size to play an important role in genetic divergence (e.g., Spurgin et al., 2014). Alternatively, increased genetic differentiation could be due to the rapid adaptation of an isolated population to novel habitat conditions. However, given that the environmental conditions seem to be similar between *alticola* and *azuay*, and *amazilia*, *caeruleigularis*, and *leucophoea*, this hypothesis seems less plausible in our study species.

## 5 CONCLUSIONS

The divergence of the six subspecies of *A*. *amazilia* has likely been shaped by a north to the south pattern of range expansion and subsequent, incomplete geographical isolation. Both genetic and geographic patterns are consistent with an expansion of the ancestral *A*. *amazilia* from the highlands and lowlands of western Ecuador into the dry environments of the Tumbesian region and central Peruvian coast. This range expansion was then likely followed by the secondary divergence of *amazilia* and *caeruleigularis* due to geographic isolation. Strong genetic evidence points to past or current introgression and gene flow among the three genetically similar subspecies of *alticola*, *dumerilii*, and *leucophoea*. Of the six subspecies, we know that *azuay* is (1) genetically most distinct from our analyses, (2) is geographically isolated in the Yunguilla Valley of Ecuador (Krabbe & Ridgely, 2010), and (3) has a distinct ventral coloration pattern with little to no rufous on the belly (Krabbe & Ridgely, 2010), all of which suggests that it could be considered at the rank of a full species. We would suggest that, if elevated to full species status, the name for this taxa should be *Amazilis azuay*. Overall, our results show that the *A*. *amazilia* subspecies are recently diverged and are in the early stages of the speciation process, as indicated by the incomplete lineage sorting of mtDNA, evidence of introgression and gene flow across multiple subspecies, eco‐climatic differentiation, and phenotypic distinctiveness, demonstrating that speciation itself is a detailed and complex process.

## CONFLICT OF INTEREST

The authors have no conflict of interest to declare.

## AUTHOR CONTRIBUTION

**Sarah A. Cowles:** Conceptualization (lead); Formal analysis (lead); Funding acquisition (supporting); Investigation (lead); Resources (equal); Supervision (lead); Writing – original draft (lead); Writing – review & editing (lead). **Christopher C. Witt:** Funding acquisition (supporting); Resources (equal); Writing – review & editing (supporting). **Elisa Bonaccorso:** Formal analysis (supporting); Resources (equal); Writing – review & editing (supporting). **Felix Grewe:** Formal analysis (supporting); Writing – review & editing (supporting). **J. Albert C. Uy:** Conceptualization (supporting); Funding acquisition (lead); Resources (equal); Supervision (supporting); Writing – review & editing (supporting).

## Supporting information

Table S1

Supplementary Material

## Data Availability

The datasets used in this study are archived in the Dryad Data Repository: https://doi.org/10.5061/dryad.cvdncjt6g.
