# Supplementary material for: Early stages of speciation with gene flow in the Amazilia Hummingbird (Amazilis amazilia) subspecies complex of Western South America
Source: Ecol Evol. 2022 May 13;12(5):e8895. doi: 10.1002/ece3.8895 (PMC9102506; doi:10.1002/ece3.8895)
Supplement: Supplementary file 2 — Supplementary Material [file ECE3-12-e8895-s002.pdf]

APPENDIX MATERIAL

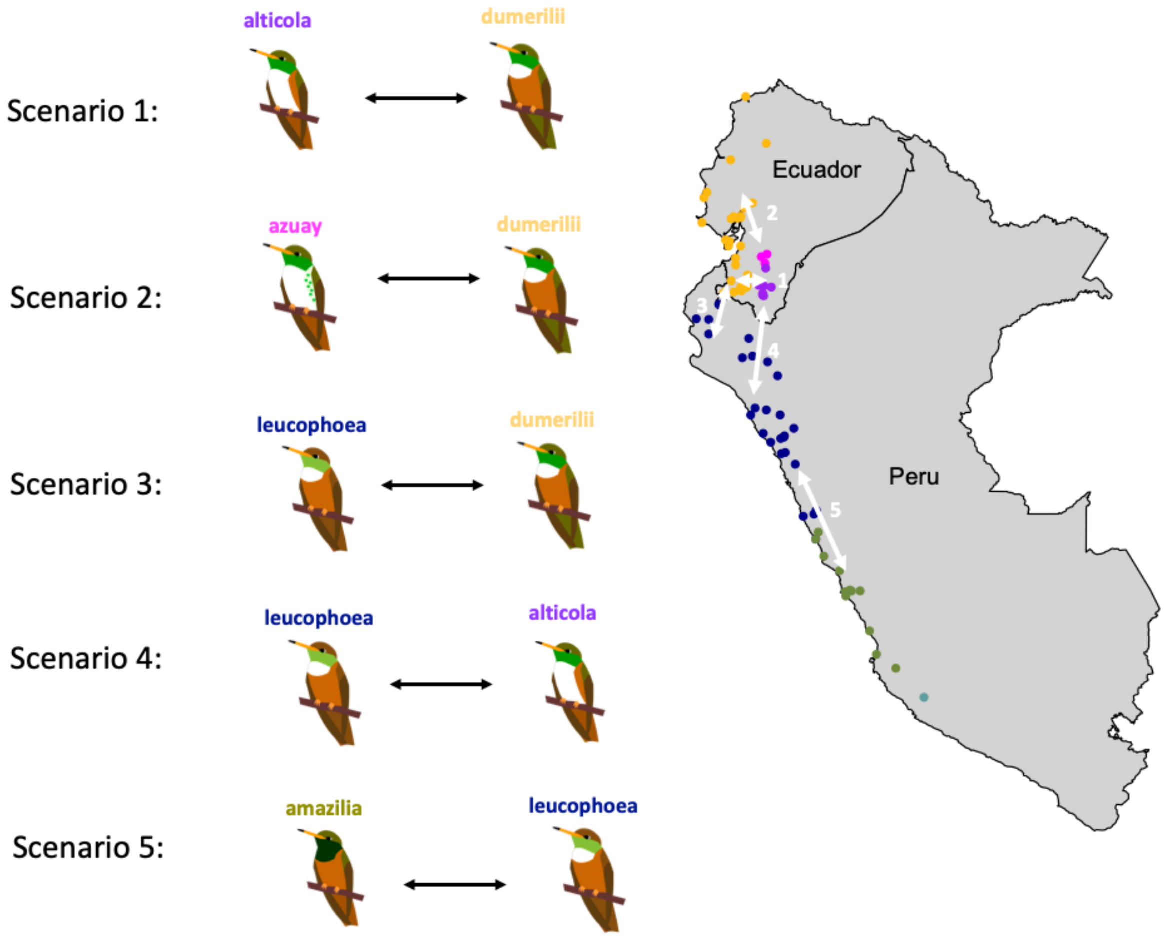

Appendix Figure 1: Five possible gene flow/introgression scenarios. These scenarios are based on geography and the phylogenetic tree (pairs could not be sister taxa or derived from one another).

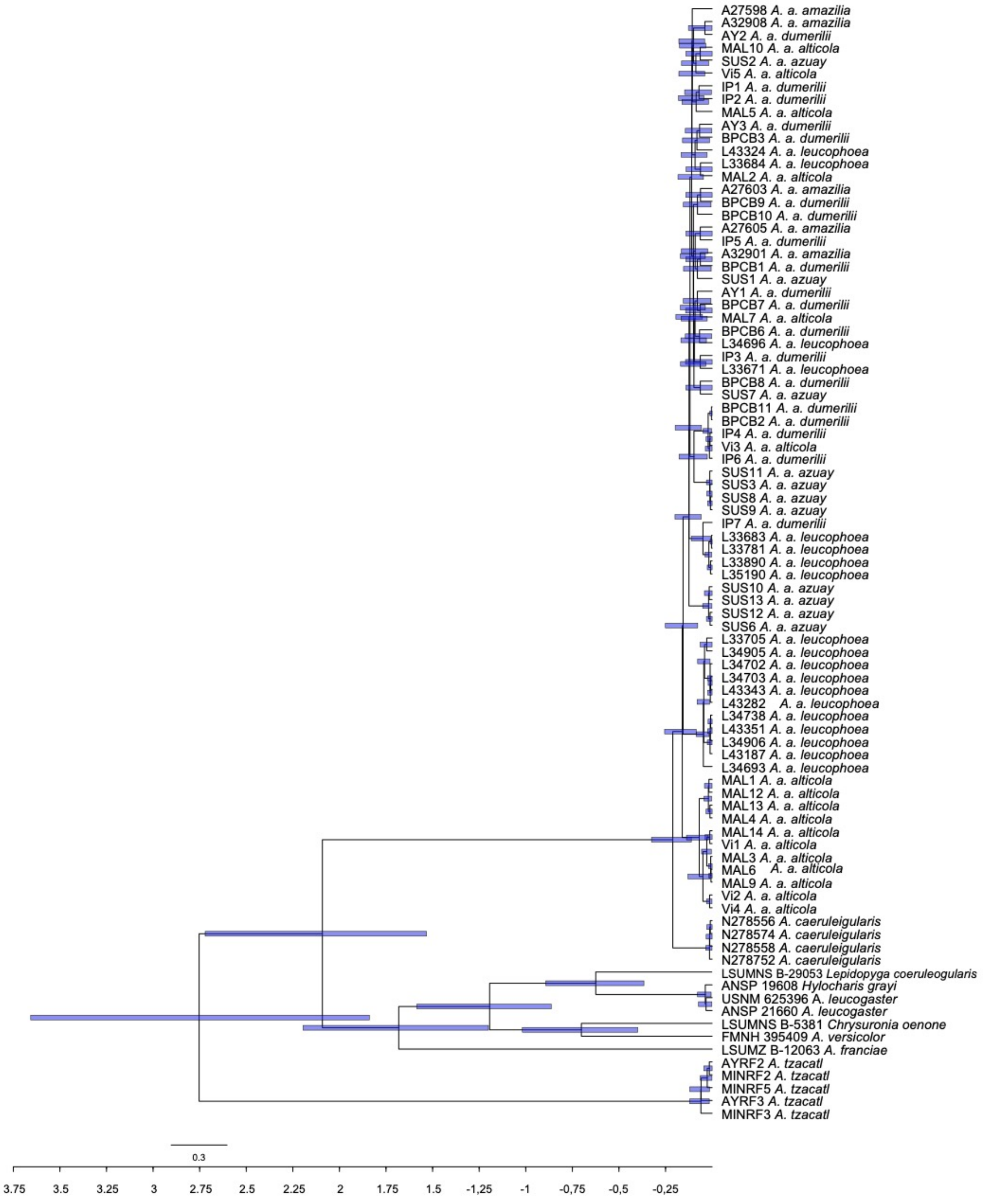

Appendix Figure 2: Maximum clade credibility tree of the mtDNA gene *ND2* obtained in BEAST. Based on ND2 sequences of *Amazilis amazilia*, congeners, and related hummingbird taxa. Time axis is based on the 0.029 substitutions/site/million year rate of Lerner et al. (2011).

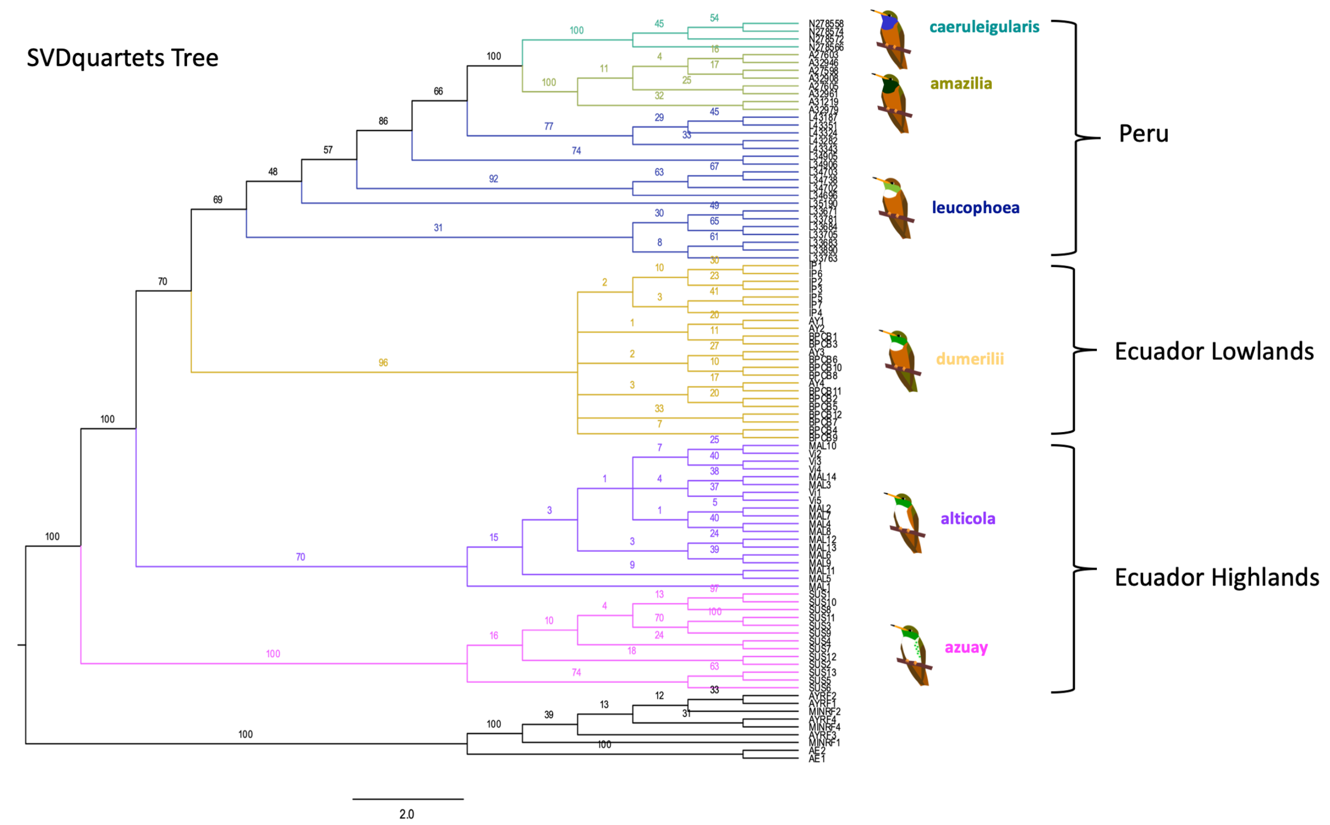

Appendix Figure 3: SVDquartets tree. The SVDquartets tree for the 86 *A. amazilia* samples and, 7 *Amazilia tzacatl,* and 2 *Uranomitra franciae*. Colors highlight different *A. amazilia* subspecies; outgroup species are colored in black. Values at branches indicate bootstrap support values.

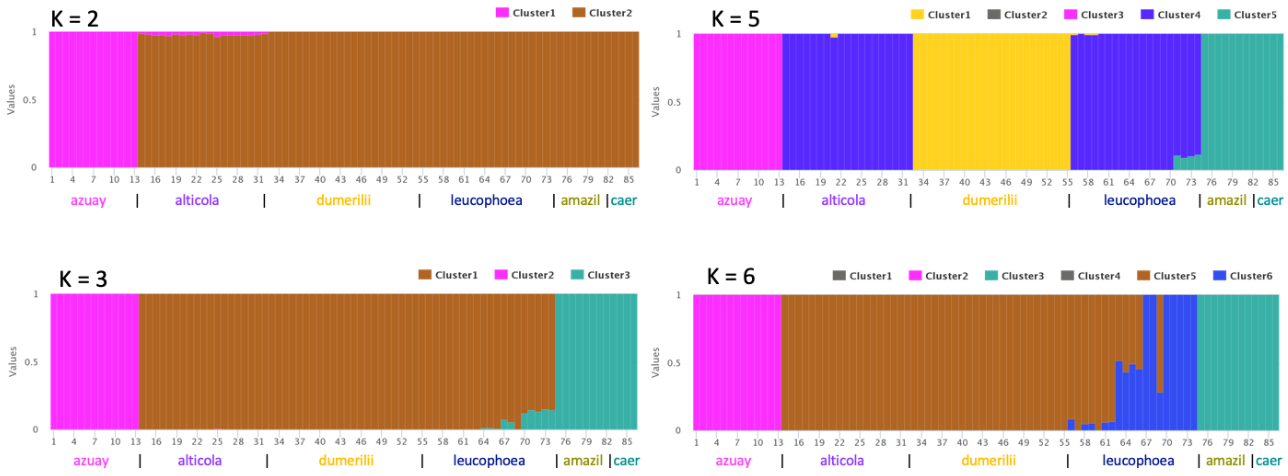

Appendix Figure 4: fastStructure plots for K = 2, 3, 5, and 6. The plot for the best-fit model, K=4, is in the main text.

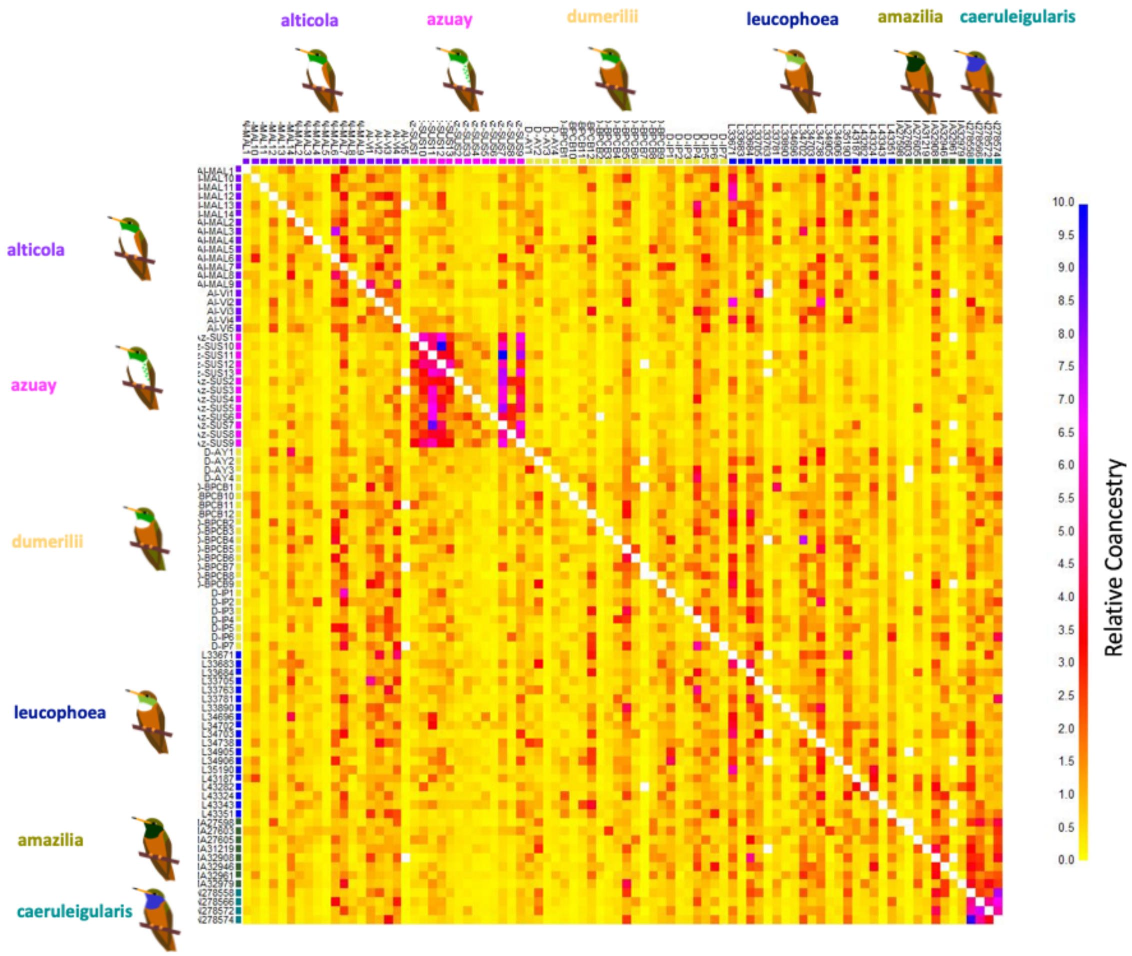

Appendix Figure 5: fineRADstructure coancestry pairwise plot for individuals organized by subspecies. Colors on heatmap represent strength of relative coancestry (blue = high, yellow = low) between pairs of individuals. Colors next to sample names identify subspecies. Only individuals from *azuay* and *caeruleigularis* showed elevated coancestry levels and subspecies clustering.

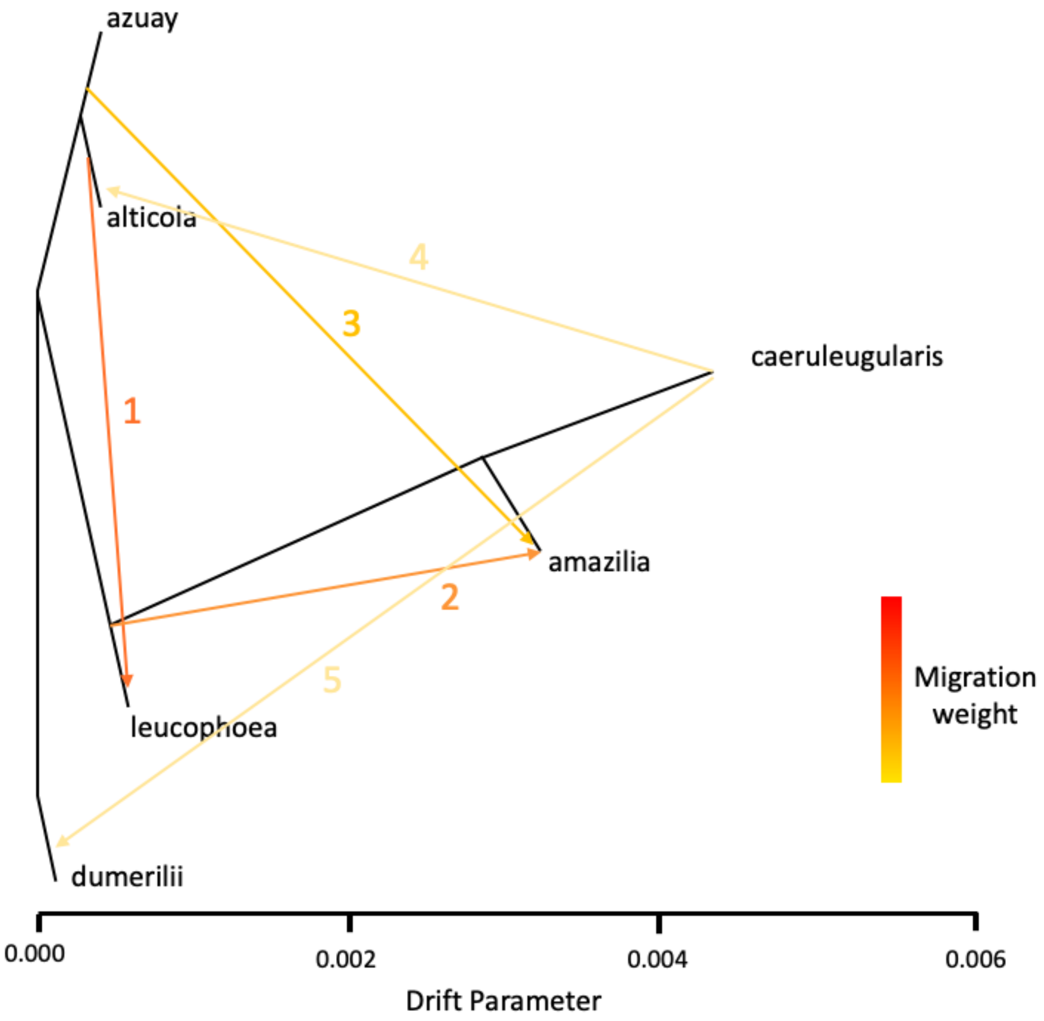

Appendix Figure 6: TreeMix results for five possible migration events. We used the RAxML tree as an input file to specify tree topology (tree is outlined in black). Colored arrows represent migration weight (red = strongest, yellow = weakest), and numbers represent ordinal number of the most likely migration events (i.e. 1-5) specified by the TreeMix program. Only scenarios 1) *alticola* to *leucophoea* and 2) *leucophoea* to *amazilia* have high migration edge weights (0.37 and 0.29, respectively; all other scenarios $\leq$ 0.07) and are in concordance with geography (i.e. subspecies that could contact one another). 3- and 4-population statistical tests also lend support to both scenarios 1 and 2 (see text for details).

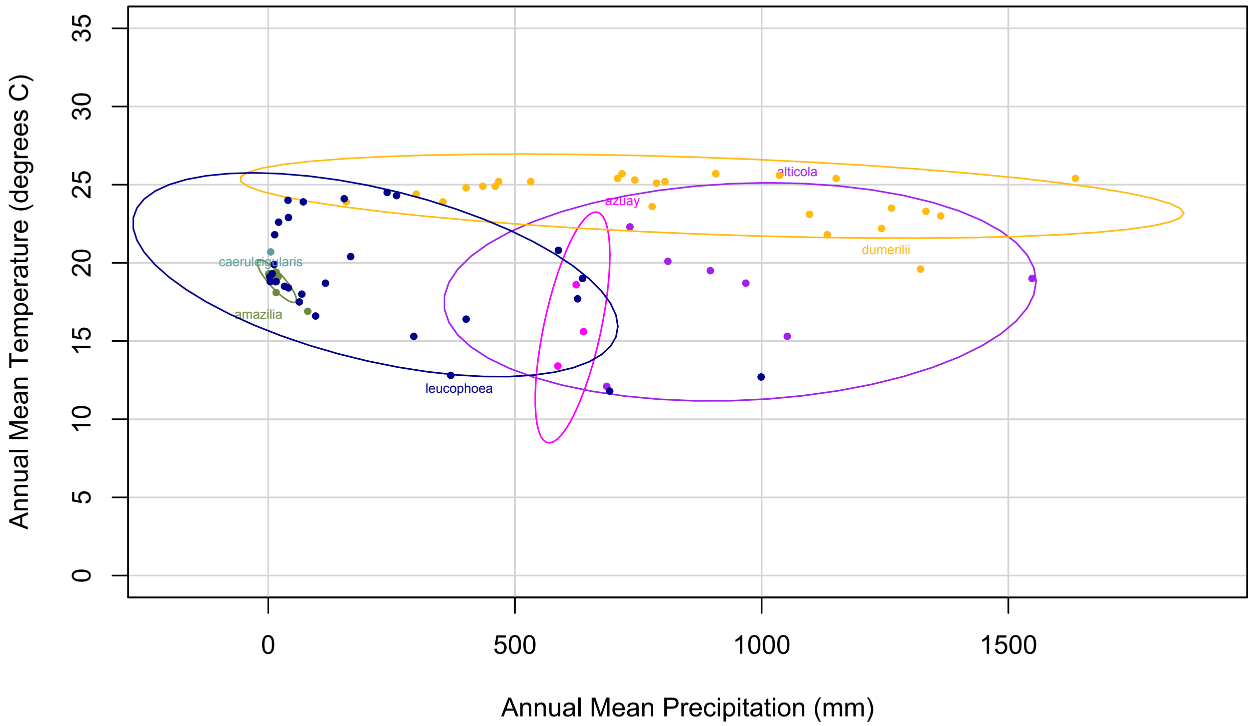

Appendix Figure 7: Annual mean temperature in ºC vs. annual precipitation in mm for each of the subspecies of *A. amazilia*. Ellipses are drawn at 80% confidence intervals for visualization.

Appendix Table 1: Details of Ecuador and Peru *Amazilis amazilia* samples

(Excel table attachment)

Appendix Table 2: Additional *ND2* sequences used in the mtDNA alignment.

**Sample ID**	**Species**	**Country**	**Province**	**GenBank Accession number**
USNM 625396 (NMNH b11307)	*Amazilia (brevirostris) leucogaster*	Guyana	Mahaica-Berbice	KJ602162
LSUMZ B-12063	*Amazilia franciae*	Ecuador	Pichincha	EU042521
ANSP (8392) 21660	*Amazilia leucogaster*	Guyana	Mahaica-Berbice	KJ602170
FMNH 395409	*Amazilia versicolor*	Brazil	Sao Paulo	EU042525
LSUMNS B-5381	*Chrysuronia oenone*	Peru	San Martín	AY830472
ANSP (5064) 19608	*Hylocharis grayi*	Ecuador	Imbabura	EU042563
LSUMNS B-29053	*Lepidopyga coeruleogularis*	Panama	-	AY830497

USNM, United States National Museum (NMNH, National Museum of Natural History); LSUMZ, Louisiana State University, Museum of Zoology; ANSP, Academy of Natural Sciences of Philadelphia at Drexel University. Information in parentheses are previous names or tissue numbers published on GenBank or previous papers.

Appendix Table 3: Estimated time since divergence of *A. amazilia* and its two closely-related relatives *Uranomitra franciae* and *Amazilia tzacatl* using maximum clade credibility trees in BEAST.

**Clade or common ancestor**	**Molecular rate (substitutions/site/million year)**		
	**0.029**	**0.0125**	**0.0068**
Amazilis amazilia	0.211 my<br>(95% HPD 0.111–0.325 my)	0.492 my<br>(95% HPD = 0.266–0.742 my)	0.910 my<br>(95% HPD =– my)
A. a. caeruleigularis	0.014 my<br>(95% HPD 0.0009–0.0340 my)	0.034 my<br>(95% HPD = 0.003–0.080 my)	0.059 my<br>(95% HPD = 0.004–0.145 my)
Common ancestor between *A. amazilia* and *Uranomitra franciae*	2.092 my<br>(95% HPD 1.533–2.720 my)	4.785 my<br>(95% HPD = 3.478–6.246 my)	11.633 my<br>(95% HPD = 7.175–16.053 my)
Common ancestor between *A. amazilia* and *Amazilia tzacatl*	2.753 my<br>(95% HPD 1.839–3.657 my)	6.336 my<br>(95% HPD = 4.320–8.519 my)	11.733 my<br>(95% HPD = 7.962–15.931 my)

Appendix Table 4: D-statistics and Bonferroni-corrected P-values for geographically possible ABBA-BABA subspecies trios (see Supplementary Fig 2 for scenarios). Introgression is directional from P3 to P2.

**Scenario**	**P1**	**P2**	**←P3**	**D-statistic**	**p-val (bonferroni corrected)**
1	*amazilia*	*alticola*	*dumerilii*	0.077	<0.0001
1	*azuay*	*alticola*	*dumerilii*	0.019	0.514
1	*caeruleigularis*	*alticola*	*dumerilii*	0.089	<0.0001
1	*leucophoea*	*alticola*	*dumerilii*	0.015	0.023
2	*amazilia*	*azuay*	*dumerilii*	0.06	0.00025
2	*caeruleigularis*	*azuay*	*dumerilii*	0.072	<0.0001
3	*amazilia*	*leucophoea*	*dumerilii*	0.067	<0.0001
3	*azuay*	*leucophoea*	*dumerilii*	0.003	1
3	*caeruleigularis*	*leucophoea*	*dumerilii*	0.079	<0.0001
4	*amazilia*	*leucophoea*	*alticola*	0.087	<0.0001
4	*azuay*	*alticola*	*leucophoea*	0.015	1
4	*caeruleigularis*	*leucophoea*	*alticola*	0.101	<0.0001
5	*caeruleigularis*	*amazilia*	*leucophoea*	0.058	<0.0001

Appendix Table 5: Dunn test statistics for pairwise comparisons between subspecies using annual mean temperature and annual precipitation. All p-values have been Bonferroni-corrected for multiple comparisons.

**TEMPERATURE**	***alticola***	***amazilia***	***azuay***	***caeruleigularis***	***dumerilii***
amazilia	Z = -0.08<br>P = 1				
azuay	Z = 0.97<br>P = 1	Z = 1.1<br>P = 1			
caeruleigularis	Z = -0.67<br>P = 1	Z = -0.65<br>P = 1	Z = -1.2<br>P = 1		
dumerilii	Z = -3.48<br>**P < 0.001**	Z = -4.6<br>**P < 0.001**	Z = -3.78<br>**P = 0.0012**	Z = -0.9<br>P = 1	
leucophoea	Z = -0.4<br>P = 1	Z = -0.38<br>P = 1	Z = -1.38<br>P = 1	Z = 0.53<br>P = 1	Z = 5.45<br>**P < 0.001**
**PRECIPITATION**	***alticola***	***amazilia***	***azuay***	***caeruleigularis***	***dumerilii***
amazilia	Z = 4.67<br>**P < 0.001**				
azuay	Z = 0.93<br>P = 1	Z = -2.45<br>P = 0.11			
caeruleigularis	Z = 2.36<br>P = 0.14	Z = 0.30<br>P = 1	Z = 1.64<br>P = 0.76		
dumerilii	Z = 0.48<br>P = 1	Z = -5.84<br>**P < 0.001**	Z = -0.72<br>P = 1	Z = -2.28<br>P = 0.17	
leucophoea	Z = 3.58<br>**P = 0.003**	Z = -2.04<br>P = 0.31	Z = 1.44<br>P = 1	Z = -1<br>P = 1	Z = 4.9<br>**P < 0.001**

Appendix Table 6: PCA loadings for all 19 Worldclim variables for PC1-PC5.

Worldclim2 Variable	PC1	PC2	PC3	PC4	PC5
**Annual Mean Temp**	-0.34	-0.12	0.01	0.09	0.04
**Mean Diurnal Range**	0.12	0.13	0.56	0.27	0.002
**Isothermality**	-0.06	0.34	0.27	-0.08	0.18
**Temp Seasonality**	0.10	-0.35	-0.09	0.20	-0.23
**Max Temp Warmest Month**	-0.29	-0.21	0.10	0.27	0.01
**Min Temp Coolest Month**	-0.34	-0.08	-0.16	-0.03	0.10
**Temperature Annual Range**	0.19	-0.15	0.40	0.40	-0.15
**Mean Temp Wettest Quarter**	-0.31	-0.14	0.12	0.13	-0.04
**Mean Temp Driest Quarter**	-0.31	-0.13	-0.12	0.09	0.11
**Mean Temp Warmest Quarter**	-0.3	-0.22	-0.02	0.15	-0.03
**Mean Temp Coldest Quarter**	-0.35	-0.04	0.02	0.04	0.09
**Annual Precipitation**	-0.18	0.34	0.02	0.02	-0.07
**Precipitation Wettest Month**	-0.23	0.28	0.15	-0.12	-0.07
**Precipitation Driest Month**	0.002	0.29	-0.27	0.47	-0.26
**Precipitation Seasonality**	-0.16	-0.12	0.45	-0.04	-0.11
**Precipitation Wettest Quarter**	-0.23	0.29	0.10	-0.12	-0.06
**Precipitation Driest Quarter**	-0.005	0.31	-0.26	0.44	-0.25
**Precipitation Warmest Quarter**	-0.22	0.20	0.06	-0.20	-0.51
**Precipitation Coldest Quarter**	-0.3	0.23	-0.03	0.33	0.66
